# Adenylate kinase hCINAP determines self-renewal of colorectal cancer stem cells by facilitating LDHA phosphorylation

**DOI:** 10.1038/ncomms15308

**Published:** 2017-05-18

**Authors:** Yapeng Ji, Chuanzhen Yang, Zefang Tang, Yongfeng Yang, Yonglu Tian, Hongwei Yao, Xi Zhu, Zemin Zhang, Jiafu Ji, Xiaofeng Zheng

**Affiliations:** 1 State Key Laboratory of Protein and Plant Gene Research, School of Life Sciences, Peking University, Beijing 100871, China; 2 Department of Biochemistry and Molecular Biology, School of Life Sciences, Peking University, Beijing 100871, China; 3 Biodynamic Optical Imaging Center, School of Life Sciences, Peking University, Beijing 100871, China; 4 Department of Critical Care Medicine, The Third Hospital, Peking University, Beijing 100871, China; 5 Department of Gastrointestinal Surgery, Key Laboratory of Carcinogenesis and Translational Research, Peking University Cancer Hospital & Institute, Beijing 100142, China

## Abstract

Targeting the specific metabolic phenotypes of colorectal cancer stem cells (CRCSCs) is an innovative therapeutic strategy for colorectal cancer (CRC) patients with poor prognosis and relapse. However, the context-dependent metabolic traits of CRCSCs remain poorly elucidated. Here we report that adenylate kinase hCINAP is overexpressed in CRC tissues. Depletion of hCINAP inhibits invasion, self-renewal, tumorigenesis and chemoresistance of CRCSCs with a loss of mesenchymal signature. Mechanistically, hCINAP binds to the C-terminal domain of LDHA, the key regulator of glycolysis, and depends on its adenylate kinase activity to promote LDHA phosphorylation at tyrosine 10, resulting in the hyperactive Warburg effect and the lower cellular ROS level and conferring metabolic advantage to CRCSC invasion. Moreover, hCINAP expression is positively correlated with the level of Y10-phosphorylated LDHA in CRC patients. This study identifies hCINAP as a potent modulator of metabolic reprogramming in CRCSCs and a promising drug target for CRC invasion and metastasis.

## Abstract (web summary)

Targeting the specific metabolic phenotypes of colorectal cancer stem cells (CRCSCs) is a potential therapeutic strategy for colorectal cancer (CRC). Here, the authors show that adenylate kinase hCINAP is overexpressed in CRC, binds to the C-terminal domain of LDHA and its depletion inhibits invasion, self-renewal, tumorigenesis and chemoresistance of CRCSCs.

Colorectal cancer (CRC) is the second most commonly diagnosed cancer in males and third most commonly diagnosed cancer in females in the Western world^1^. Liver metastasis is the primary reason for colorectal cancer mortality^2^; the 5-year survival rate of CRC patients with metastases is just 13% (American Cancer Society. Cancer Facts & Figures 2016). There is emerging evidence that cancer stem cells (CSCs), also known as tumour-initiating cells (TICs), are the primary cells responsible for the seeding and colonization of distant metastases^3^. CSCs are tumour cells possessing the properties of self-renewal, multilineage differentiation, clonal tumour initiation and sustenance capacity and distant repopulation potential^4^. The CSC hypothesis suggests that, like normal colorectal tissues, CRC cells are organized hierarchically and depend on CSCs for population maintenance^5^. Poor prognosis and relapse have been attributed to CSCs^6^. Thus, targeting colorectal cancer stem cells (CRCSCs) may be an effective treatment strategy. CRCSCs are CD133^+^ cells capable of *in vitro* and *in vivo* tumour propagation^7^. Several CRCSC markers have been characterized, including LGR5 (ref. 8), ALDH1 (ref. 9) and CD44 (ref. 10). Epithelial cancer cells, including CRCSCs, often undergo the epithelial-to-mesenchymal transition (EMT), during which they lose cell–cell adhesion and cellular polarity, remodel the CSC microenvironment by degrading the extracellular matrix (ECM) and basement membrane, acquire stem cell-like, migratory and invasive properties, and develop resistance to apoptosis and chemotherapy^11^, with downregulation of epithelial cadherin (E-cadherin) and upregulation of *TWIST1*, *Zeb1*, *Snail1*, *vimentin* and neural cadherin (N-cadherin)^12^. The CSC population in tumours increases after chemotherapy, indicating a connection between CSCs undergoing the EMT and resistance to therapeutic agents^13^; indeed, in CRC patients, EMT activation is associated with a poor likelihood of survival^14^.

A common feature of cancer cell metabolism is the ability to acquire necessary nutrients from a nutrient-poor environment and utilize these nutrients to maintain viability and build new biomass. One of the principal distinguishing characteristics of tumour cells is the switch from oxidative phosphorylation to glycolysis as an energy supply, even when sufficient oxygen is present; this change is known as the Warburg effect or aerobic glycolysis^15^. Enhanced glycolysis produces intermediate metabolites, especially nucleic acids and fatty acids, required for biosynthetic pathways and rapid proliferation^16^. The Warburg effect has been closely connected to tumour growth, invasion and CSC identity^17^. Metabolic modulation plays a crucial role in cell growth; dysregulation of many metabolic enzymes contributes to the Warburg effect and tumorigenesis^18^,^19^, including lactate dehydrogenase A (LDHA), which is frequently upregulated by c-Myc and HIF1α in various cancers^20^. Inhibition of LDHA restrains the energy supply of tumour cells and thereby reduces their capacity to proliferate and invade^21^. However, the metabolic scenario in CRCSCs has not been studied in detail.

Human coilin-interacting nuclear ATPase protein (hCINAP), also known as adenylate kinase 6 (AK6), has an adenylate kinase domain that is highly conserved in many organisms^22^,^23^. Histidine 79 of hCINAP is involved in AMP binding; an H79G hCINAP mutant showed a 75% reduction in adenylate kinase activity^24^. Fap7, a hCINAP homologue in yeast, is essential for 18S rRNA maturation^25^. It has been reported that hCINAP is indispensable for *Cajal* body formation and supports cell viability^26^,^27^.

Herein, we demonstrate that metabolic reprogramming of CRCSCs is modulated by hCINAP. We find that hCINAP is abnormally overexpressed in CRCs and markedly promotes CRC cell migration and invasion. Depletion of hCINAP attenuates a set of CRCSC phenotypes, including self-renewal, the EMT and chemoresistance. Further studies reveal that hCINAP directly binds to the C-terminal domain of LDHA, relies on its adenylate kinase activity to regenerate ATP from ADP, and facilitates Y10 LDHA phosphorylation catalysed by FGFR1, thus alleviating intracellular ROS production and conferring metabolic advantage and a microenvironmental benefit to CRCSC invasion. Intriguingly, by reinforcing the glycolytic phenotype, hCINAP enhances the resistance of CRC cells to metabolic stress caused by nutrient deficiency. These results reveal that adenylate kinase hCINAP is responsible for the energy requirements of invasive CRCSCs. Our findings provide a novel clinical target for CRC treatment and illuminate the mechanisms of CRC invasion, CRC metastasis, and metabolic reprogramming in CSCs.

## Results

### Highly expressed hCINAP promotes CRC migration and invasion

**Figure 1:**
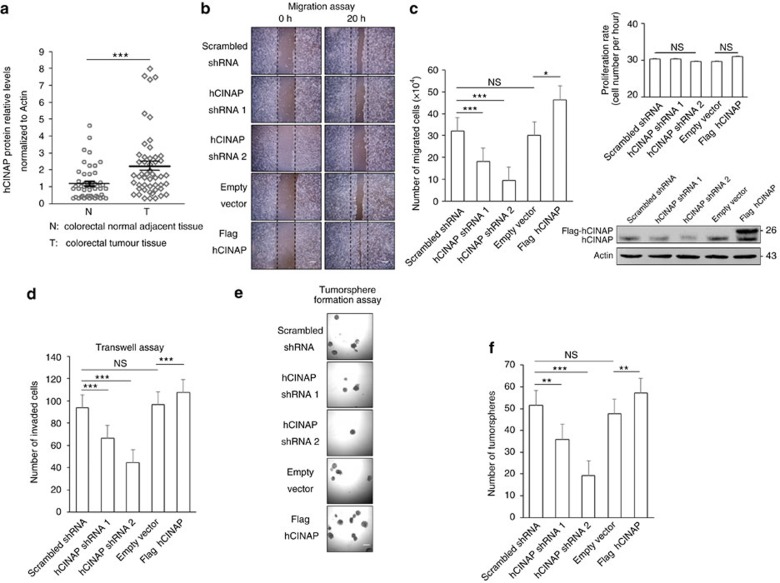
hCINAP promotes invasiveness and metastasis in CRC cells. (**a**) Western blot analysis of the hCINAP protein levels in human CRC and adjacent paired normal tissues, normalized to Actin (*n*=50). See also Supplementary Fig. 1. (**b**) The wound-healing assay using SW480 cells expressing the indicated shRNAs and plasmids. Scale bar, 100 μm. (**c**) Quantification of the migration potential and proliferation rate of the cells in the migration assay (*n*=3 wells each group) and western blot analysis of the hCINAP and Flag-hCINAP expression. (**d**) Quantification of invaded cells in the transwell assay (*n*=3). (**e**) Tumorsphere formation assay using CRCSCs transfected as indicated. Scale bar, 100 μm. (**f**) Quantification of the tumorsphere formation assay (*n*=3). Asterisks indicate significant differences (*P<0.05, **P<0.01, ***P<0.001) determined by Student's *t*-test (**a**,**c**,**d**,**f**). NS, not significant.

It has been reported that hCINAP is indispensable for cell viability and tumour cell growth^28^. We analysed hCINAP expression in colorectal cancers (CRC) based on the TCGA COAD (Colon adenocarcinoma) and READ (Rectum adenocarcinoma) cancer types, and found that the level of hCINAP protein was significantly higher in tumour tissue than that in adjacent non-tumour tissue (Supplementary Fig. 1d). Consistently, hCINAP protein was significantly upregulated in CRC tissues from 50 patients in comparison with its level in adjacent normal tissues (Fig. 1a; Supplementary Table 2; Supplementary Fig. 1a,b). Immunohistochemical analysis of hCINAP expression identified the heterogeneity of CRC (Supplementary Fig. 1c). We also investigated hCINAP expressions in different CRC stages and molecular subtypes based on a four consensus molecular subtypes (CMSs) classification^29^. No significant difference of hCINAP levels was observed in four CRC stages (Supplementary Fig. 1e), while the hCINAP level was relatively higher in CMS2 with marked activation of Wnt and Myc signalings, and CMS3 with disordered metabolism (Supplementary Fig. 1f). Hyperactive Wnt signalling and metabolic dysregulation are frequently revealed in CRC^30^,^31^ and Wnt pathway contributes to CRCSCs self-renewal and metastasis^32^. As metastasis is the primary cause of mortality in patients with CRC, we assessed whether hCINAP affected CRC cell migration and invasion. hCINAP-depleted SW480 cells showed impaired migration potential in a wound-healing assay, while overexpressing hCINAP rendered SW480 cells more migratory (Fig. 1b). Of note, cells transfected with more effective shRNA showed worse migration (Fig. 1c). In addition, transwell assays showed that hCINAP silencing markedly impeded cell invasion, whereas hCINAP overexpression promoted SW480 cell invasion (Fig. 1d), suggesting that aberrant overexpression of hCINAP expedited CRC cell motility.

CRCSCs, also known as CR tumour-initiating cells (CRTICs), are a cohort of stem cell-like tumour cells with invasive and metastatic capacities. We investigated the role of hCINAP in promoting TIC characteristics. Indeed, treatment with shRNA against hCINAP inhibited tumorsphere formation by tumour cells isolated from liver metastases of CRC patients (Supplementary Fig. 5a), while overexpression of hCINAP significantly enhanced tumorsphere formation by these cells (Fig. 1e,f).

### hCINAP is required for CRCSC-associated phenotypes

**Figure 2:**
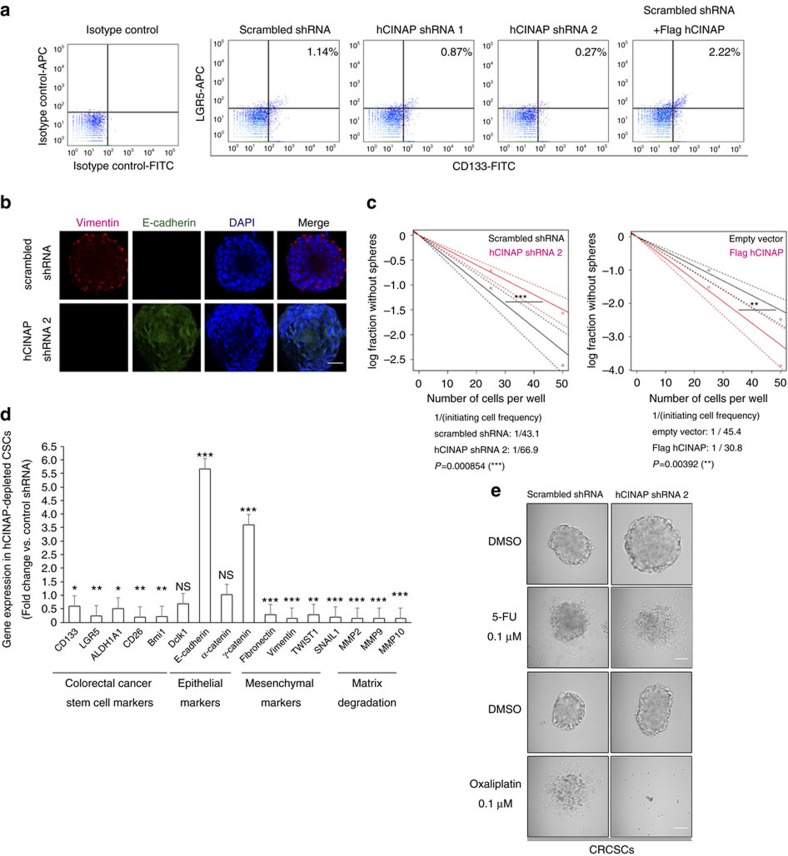
hCINAP determines CRCSC phenotypes in CRC cells. (**a**) FACS plots of CD133 and LGR5 staining in patient-derived colorectal cancer stem cells (CRCSCs) expressing the indicated shRNAs and plasmids. See also molecularly characterized CRCSCs derived from patient in Supplementary Fig. 5a. (**b**) Immunofluorescence analysis of the effect of hCINAP knockdown by shRNA on the mesenchymal–epithelial transition in CRCSC spheres. Mesenchymal marker vimentin was labelled in red and epithelial marker E-cadherin was labelled in green. The nucleus was counterstained with DAPI (blue). Scale bar, 100 μm. (**c**) Limiting dilution tumorsphere formation assay in CRCSCs with the indicated shRNAs and plasmids. CRCSC frequency was calculated using extreme limiting dilution analysis. **P<0.01, ***P<0.001. (**d**) Representative mRNA levels were analysed by qRT–PCR in CRCSCs as indicated. Gene expression in hCINAP-depleted cells was normalized to that of the shRNA control group. The experiments were performed three times and data are determined by Student's *t*-test and presented as mean±s.e.m. (*P<0.05, **P<0.01, ***P<0.001, NS, not significant). (**e**) CRCSC spheres were treated with 0.1 μM 5-fluorouracil or 0.1 μM oxaliplatin for 24 h and photographed using a bright field microscope in **e**. Scale bar, 50 μm. Also see that the viable cell inhibition ratio was determined using MTT colorimetry in Supplementary Fig. 6c.

CRCSCs are capable of self-renewal, tumour initiation and long-term repopulation; as a result of these characteristics, CRCSCs are associated with aggressivity, poor prognosis and relapse^33^. To assess the role of hCINAP in CRCSC maintenance, CRCSC tumorspheres derived from CRC liver metastases were transfected with control shRNA, hCINAP shRNA 1, hCINAP shRNA 2 and Flag-hCINAP, respectively, after which they were subjected to fluorescence-activated cell sorting (FACS) analysis. CRCSC markers CD133 and LGR5 were upregulated in hCINAP-overexpressing cells, whereas hCINAP RNAi knockdown reduced expression of CD133 and LGR5 (Fig. 2a). Epithelial cancer cells are inclined to undergo the EMT. Indeed, immunofluorescence analysis showed that hCINAP depletion enhanced expression of epithelial marker E-cadherin and reduced expression of mesenchymal marker vimentin (Fig. 2b), suggesting that hCINAP promoted induction of the EMT in CRC cells. Furthermore, we tested whether hCINAP played a role in CRCSC self-renewal using a limiting dilution assay *in vitro*. hCINAP silencing significantly decreased the proportion of self-renewing sphere-forming units, while overexpression of hCINAP promoted tumorsphere formation by self-renewing CRCSCs (Fig. 2c). In addition, quantitative PCR with reverse transcription (qRT–PCR) showed that hCINAP depletion reduced expression of CRCSC markers such as CD133 and LGR5, epithelial markers such as E-cadherin and γ-catenin, and CSC niche remodellers matrix metalloproteinase 2, matrix metalloproteinase 9 and matrix metalloproteinase 10. In contrast, knockdown of hCINAP promoted expression of mesenchymal markers (Fig. 2d). It has been well-established that metastatic CRCSCs are resistant to chemotherapy^34^; therefore, we assessed whether hCINAP affected the sensitivity of CRCSC spheres to chemotherapeutic drugs. Treatment with hCINAP shRNA 2 followed by 5-FU or oxaliplatin increased cell death in tumorspheres (Fig. 2e; Supplementary Fig. 6c), suggesting that hCINAP contributes to chemoresistance of colorectal tumour cells. On the basis of these observations, we conclude that hCINAP overexpression is crucial for self-maintenance and self-renewal by CRCSCs.

### hCINAP directly binds to LDHA and enhances LDHA activity

**Figure 3:**
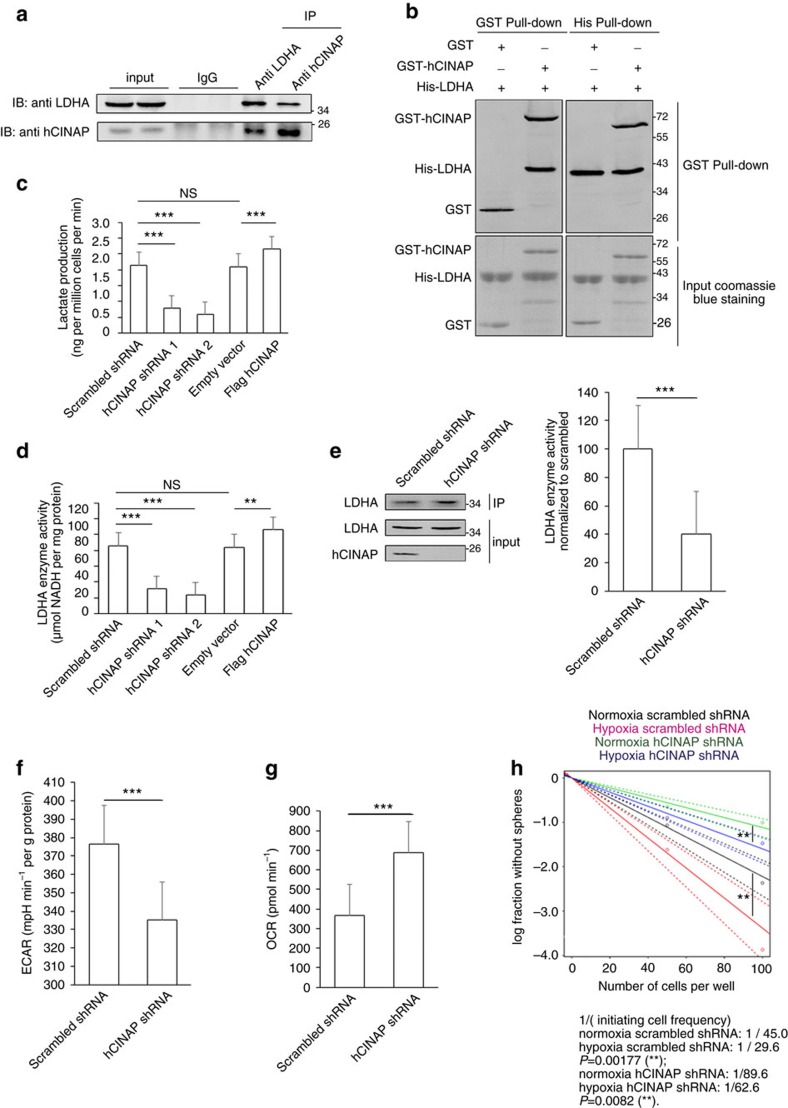
hCINAP directly binds to LDHA and promotes LDHA activity. (**a**) Immunoprecipitation analysis of the interaction between endogenous hCINAP and LDHA protein in SW480 cells. Rabbit IgG served as a negative control. See also the hCINAP-interacting partners screening in Supplementary Fig. 2. (**b**) *In vitro* pull-down analysis of the interaction between GST-tagged hCINAP and His-tagged LDHA protein purified from *E. coli*, with GST protein as a negative control. See also the details within the FGFR1–LDHA–hCINAP interaction in Supplementary Fig. 3. (**c**) Lactate production levels in SW480 cells transfected with the indicated shRNAs and plasmids were determined by a spectrophotometer and normalized to the cell number. (**d**) LDHA enzyme activity was determined in SW480 cellls as indicated. (**e**) Endogenous LDHA protein was immunoprecipitated and subjected to enzymatic activity assay. Western blot analysis showed immunoprecipitated LDHA protein in lysates of SW480 cells treated with control shRNA and hCINAP shRNA 2. The experiments were performed three times. (**f**,**g**) Measurements of ECAR and OCR levels of SW480 cells in **e**. (**h**) Limiting dilution assay in CRCSCs as indicated under normaxia or hypoxia (3% O_2_). CRCSC frequency is calculated by extreme limiting dilution analysis. **P<0.01. Also see LDHA Y10 phosphorylation and LDHA activity in Supplementary Fig. 6a,b. Data in **c**–**g** are determined by Student's *t*-test and presented as mean±s.e.m. (*n*=3). **P<0.01, ***P<0.001, NS, not significant.

To elucidate the manner in which hCINAP functions in CRCSCs, we conducted mass spectrometry to identify hCINAP-interacting partners linked to tumour biology (Supplementary Fig. 3a,b). Proteomics analysis showed that lactate dehydrogenase A (LDHA) was a candidate interacting with hCINAP. Although hCINAP protein was distributed in both cytosol and nucleus, hCINAP in cytosol co-localized with LDHA (Supplementary Fig. 7a–c). Co-IP analyses verified that hCINAP endogenously interacted with LDHA but not LDHB (Fig. 3a; Supplementary Fig. 3a). In addition, *in vitro* GST- and His-pull-down assays showed that hCINAP directly bound to LDHA (Fig. 3b). LDHA, a pivotal metabolic enzyme in glycolysis and the Warburg effect, converts pyruvate into lactate for extracellular secretion, remodels the tumour microenvironment, and promotes tumour invasion and metastasis^35^. Accordingly, we investigated whether hCINAP influenced the enzymatic activity of LDHA. hCINAP depletion reduced the abundance of extracellular lactate, whereas hCINAP overexpression increased lactate production in SW480 cells (Fig. 3c). Moreover, hCINAP abundance was positively correlated with LDHA activity (Fig. 3d). To preclude the possibility that other cellular components, such as NAD^+^/NADH, disturbed the measurement of LDHA activity, endogenous LDHA in SW480 cells transfected with control shRNA and hCINAP shRNA 2 was immunoprecipitated and LDHA activity was measured. Indeed, hCINAP depletion impaired LDHA activity, but had no effect on LDHA expression (Fig. 3e), indicating that hCINAP affects lactate production by directly binding to LDHA and regulating LDHA activity.

In addition, hCINAP depletion reduced ECARs and augmented OCRs in CRC (Fig. 3f,g), suggesting that hCINAP promotes glycolysis and restrains oxidative phosphorylation (OXPHOS) by enhancing LDHA activity. Moreover, hypoxia is linked to cancer cell glucose metabolism, EMT and CSCs differentiation^36^, and we found that hypoxia duplicated the effect of hCINAP on self-renewal potential of CRCSCs in limited dilution assays (Fig. 3h). We also examined LDHA activities in CRCSCs treated with control or hCINAP shRNA under normoxia or hypoxia (Supplementary Fig. 6a). Still, hypoxia had additive promotion effect on LDHA activity in CRCSCs by hCINAP. However, LDHA, but not hCINAP expression, was elevated in hypoxic condition (Supplementary Fig. 6b), indicating that the promotion of LDHA activity results from the enhanced LDHA expression, instead of hCINAP, by the hypoxia-inducible protein HIF1a in CRCSCs residing in hypoxic niches^37^.

### Adenylate kinase hCINAP facilitates LDHA Y10 phosphorylation

**Figure 4:**
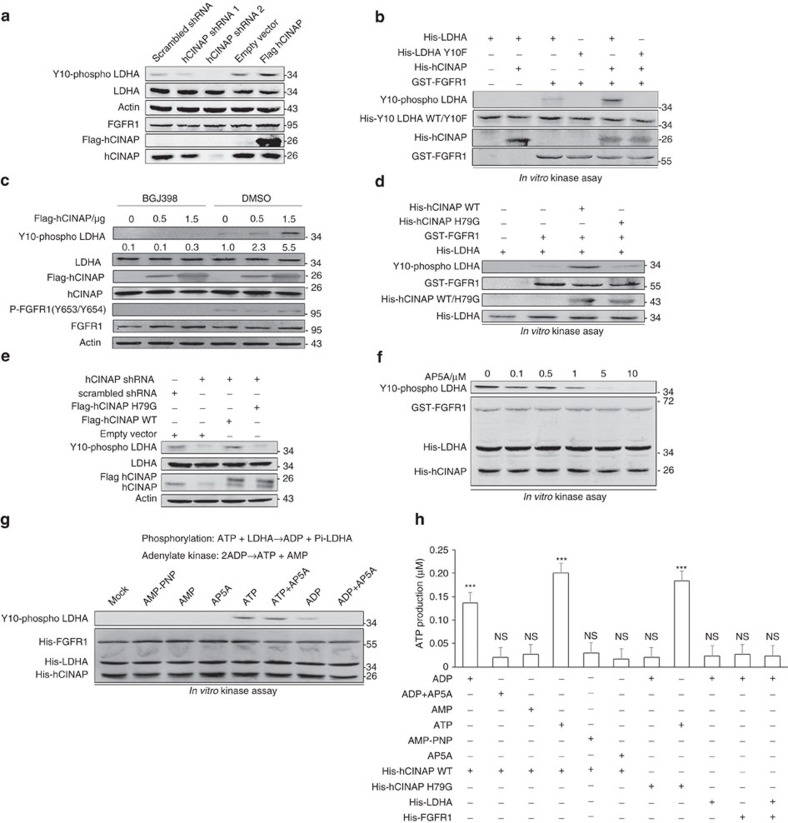
Adenylate kinase hCINAP promotes LDHA Y10 phosphorylation catalysed by FGFR1. (**a**) Western blot analysis of LDHA Y10 phosphorylation levels in SW480 cells transfected as indicated. See also hCINAP-enhanced LDHA tetramerization in Supplementary Fig. 4. (**b**) Immunoblot analysis of LDHA Y10 phosphorylation catalysed by FGFR1 *in vitro* with or without hCINAP. Y10F LDHA served as a negative control. (**c**) Western blot analysis of LDHA Y10 phosphorylation in SW480 cells transfected with the indicated plasmids and treated with FGFR1 inhibitor BGJ398 (10 nM) or DMSO. (**d**,**e**) The effects of wild-type hCINAP and H79G mutant hCINAP on FGFR1-catalysed LDHA Y10 phosphorylation *in vitro* (**d**) and in SW480 cells expressing the indicated plasmids (**e**). (**f**) The effect of AP5A on LDHA Y10 phosphorylation in FGFR1 kinase assay *in vitro*. (**g**) *In vitro* FGFR1 kinase assay with various combinations of protein and nucleotides as indicated. (**h**) ATP levels were measured by a luciferase reporter system in assays with the indicated combinations of protein and nucleotides. The results are expressed as mean±s.e.m. of three independent experiments. Asterisks indicate significant differences (***P<0.001 versus second column) determined by Student's *t*-test.

Given that LDHA activity is determined by LDHA tetramerization and the binding capacity of LDHA to NADH^38^, we tested whether hCINAP depletion influenced these factors. Gel filtration chromatography and western blot analysis showed that LDHA tetramer abundance decreased upon hCINAP RNAi knockdown (Supplementary Fig. 4a–c). However, Cibacron blue binding assays^39^ showed that hCINAP depletion had no effect on the affinity between NADH and LDHA (Supplementary Fig. 4d). These results suggest that hCINAP influences LDHA tetramerization, but not the binding capacity of LDHA for NADH. Given that LDHA tetramerization is determined by LDHA phosphorylation at tyrosine 10 catalysed by FGFR1 (ref. 38), we tested whether hCINAP-enhanced LDHA Y10 phosphorylation. As expected, hCINAP promoted LDHA Y10 phosphorylation in SW480 cells (Fig. 4a). *In vitro* FGFR1 kinase assays verified that hCINAP promoted LDHA phosphorylation by FGFR1 (Fig. 4b), rather than JAK2 or c-Abl (Supplementary Fig. 3b,c), and hCINAP did not directly phosphorylate LDHA at Y10 (Fig. 4b, lanes 1 and 2). hCINAP facilitated FGFR1-catalysed phosphorylation of wild-type LDHA, but not phosphorylation of Y10F mutant LDHA (Fig. 4b, lanes 3–6). Moreover, LDHA Y10 phosphorylation was not promoted by hCINAP in SW480 cells treated with FGFR1 inhibitor BJG398 (Fig. 4c). We found that LDHA directly bound to both hCINAP and FGFR1, thus forming the ternary protein complex, while hCINAP did not bind to FGFR1 directly (Supplementary Fig. 3d). Taken together, these results suggest that hCINAP enhances LDHA Y10 phosphorylation catalysed by FGFR1.

Notably, hCINAP possesses adenylate kinase activity and buffers drastic changes in ATP abundance by converting two ADP molecules into one ATP molecule and one AMP molecule, thus playing a pivotal role in cellular energy homeostasis^40^. To elucidate whether hCINAP relies on its own enzymatic activity to influence LDHA phosphorylation, we performed *in vitro* FGFR1 kinase assays using wild-type hCINAP and H79G mutant hCINAP with impaired adenylate kinase activity. As expected, H79G mutant hCINAP did not promote LDHA Y10 phosphorylation (Fig. 4d), although H79G mutant had no impact on the LDHA–hCINAP interaction (Supplementary Fig. 3e). In addition, hCINAP depletion in SW480 cells attenuated LDHA Y10 phosphorylation; ectopic expression of wild-type hCINAP, but not H79G mutant hCINAP, restored the LDHA Y10 phosphorylation level in hCINAP-depleted SW480 cells (Fig. 4e). These results indicate that the adenylate kinase activity of hCINAP is vital for LDHA phosphorylation and activity.

hCINAP also possesses weak ATPase activity^24^. Generally, adenylate kinases act as weak ATPases by converting ATP and AMP to ADP. To assess whether the adenylate kinase activity of hCINAP was its most important activity with regard to promoting LDHA phosphorylation, we performed *in vitro* FGFR1 kinase assays and found that the LDHA Y10 phosphorylation inhibited by AP5A in a concentration-dependent manner (Fig. 4f), indicating that AP5A inhibits LDHA phosphorylation by inhibiting the adenylate kinase activity of hCINAP.

Next, we investigated the manner in which hCINAP facilitates LDHA phosphorylation via its adenylate kinase activity. *In vitro* FGFR1 kinase assays with the addition of hCINAP and different adenosine derivatives revealed that ATP, but not AMP, adenylyl imidodiphosphate (AMP-PNP, an non-hydrolysed ATP analogue), or AP5A, was utilized for FGFR1-catalysed LDHA phosphorylation (Fig. 4g, lanes 1–4). However, LDHA Y10 phosphorylation was detected when ADP was added (Fig. 4g, lane 7), and AP5A inhibited ADP utilization (Fig. 4g, lane 8), implying that hCINAP generated ATP from ADP for LDHA phosphorylation. Indeed, measurement of ATP production by luciferase chemiluminescence analysis showed that hCINAP produced ATP from ADP (Fig. 4h), and hCINAP promoted the recruitment of ATP to FGFR1 in kinase assays *in vitro* (Supplementary Fig. 3f). These results suggest that hCINAP facilitates LDHA phosphorylation by converting ADP to ATP.

### hCINAP confers resistance to metabolic stress in CRCs

**Figure 5:**
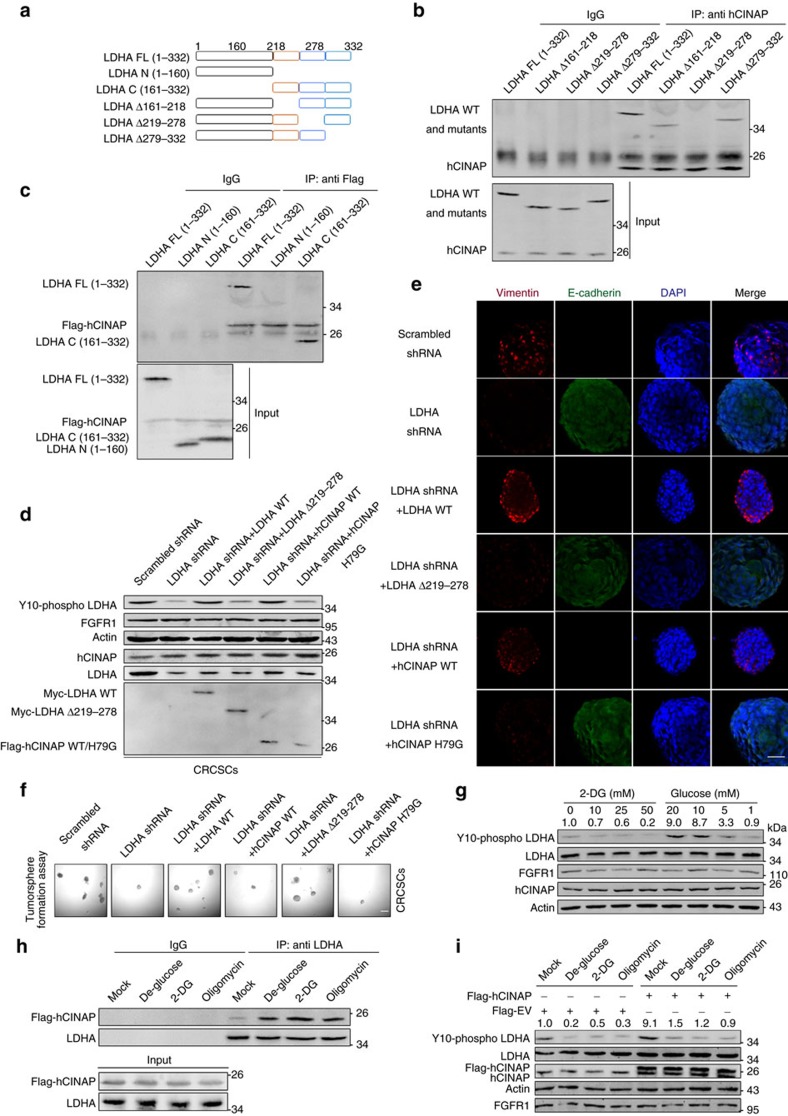
The C-terminal domain of LDHA recruits hCINAP to facilitate LDHA Y10 phosphorylation in a low-energy state. (**a**) Schematic diagram of the structural domain organization within LDHA. (**b**,**c**) Co-IP analyses of the interaction between various regions of LDHA and hCINAP in SW480 cells. IgG served as the negative control. (**d**) Western blot analysis of LDHA Y10 phosphorylation level in CRCSCs transfected with indicated plasmids. (**e**) Immunofluorescence analysis of the effect of shRNA-depleted LDHA and LDHA/hCINAP rescue expression on the mesenchymal–epithelial transition in CRCSCs labelled by Vimentin (red), E-cadherin (green). The nucleus was counterstained with DAPI (blue). Scale bar, 100 μm. (**f**) Tumorsphere formation assay using cells in **e**. Scale bar, 100 μm. (**g**) Western blot analysis of LDHA Y10 phosphorylation in CRCSCs treated with different doses of 2-DG and glucose as indicated. (**h**) Co-IP assay for the interaction between endogenous hCINAP and LDHA protein in CRCSCs without glucose supply (de-glucose) or treated with 2-DG (50 mM) or oligomycin (1 μM). (**i**) The effects of 2-DG, glucose deprivation and oligomycin on LDHA Y10 phosphorylation in CRCSCs transfected with an empty vector or Flag-tagged hCINAP.

We next mapped the critical region of LDHA responsible for its binding to hCINAP. Co-IP analysis showed that hCINAP interacted with the C-ternimal domain of LDHA, instead of its N-terminal catalytic subunit (Fig. 5a,b). In detail, the C-terminal 219–278 region was essential for the LDHA–hCINAP binding (Fig. 5c). Furthermore, we illustrated whether the direct interaction between hCINAP and LDHA was indispensable for CRCSC stemness maintenance. Knockdown of LDHA reduced LDHA phosphorylation significantly; the level of LDHA phosphorylation could only be rescued by LDHA or wild-type hCINAP but not LDHA Δ219–278 or hCINAP H79G mutant (Fig. 5d). Immunofluerescence analysis exhibited that LDHA depletion impaired the EMT program, whereas the rescue expression of wild-type LDHA or hCINAP, not LDHA Δ219–278 or hCINAP H79G mutant, restored the EMT in CRCSCs (Fig. 5e). The consistent results were obtained in tumorsphere formation assays (Fig. 5f). These data indicate that hCINAP, relying on LDHA, contributes to CRCSC stemness by directly binding to LDHA and enhancing LDHA Y10 phosphorylation via adenylate kinase activity.

The results in Fig. 4g suggest that LDHA Y10 phosphorylation is susceptible to fluctuations in the abundance of ATP and nutriture, suggesting a feedback mechanism in its regulation of LDHA activity. CRC cells require sustaining nutrients and oxygen to support growth induced by excessive proliferation signalling^41^, resulting in nutrient deficiency in primary tumours and metastatic sites, at least partially. We thus examined whether the glucose supply influenced LDHA Y10 phosphorylation. In cells treated with 2-deoxy-D-glucose (2-DG), a glucose analogue that competitively inhibits production of glucose-6-phosphate from glucose, LDHA Y10 phosphorylation was significantly inhibited (Fig. 5g, lanes 1–4). Reducing the glucose concentration led to reduced LDHA phosphorylation, but did not affect protein levels of hCINAP or FGFR1 (Fig. 5g, lanes 5–8).

Next, we investigated whether metabolic stress influenced the interaction between hCINAP and LDHA. Indeed, both glucose deprivation and 2-DG perturbation promoted hCINAP–LDHA interaction. Moreover, oligomycin, an inhibitor of ATP synthase that impedes ATP production via oxidative phosphorylation, strengthened hCINAP–LDHA interaction (Fig. 5h). These results suggest that low cellular ATP abundance decreased LDHA Y10 phosphorylation, whereas hCINAP overexpression in CRC cells allows such cells to overcome unfavourable nutritional conditions. Indeed, overexpression of hCINAP in CRC cells reversed the decrement of LDHA Y10 phosphorylation caused by glucose deprivation, 2-DG and oligomycin treatment (Fig. 5i). These results suggest that high hCINAP expression promotes resistance to adverse metabolic states in CRC cells.

### Adenylate kinase hCINAP enhances glycolysis in CRCSCs

**Figure 6:**
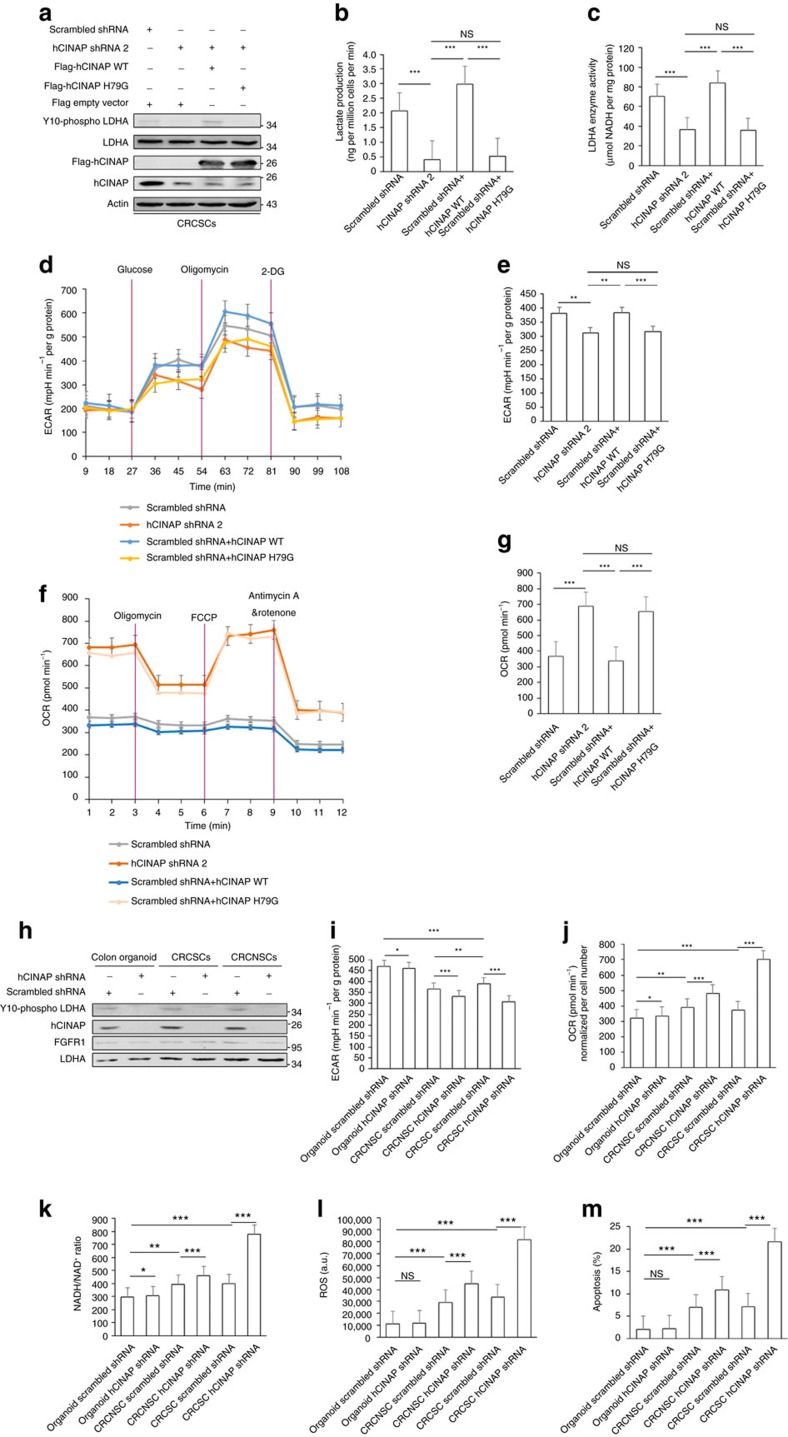
Adenylate kinase hCINAP promotes the Warburg effect in CRC cells. (**a**) Immunoblot analysis of LDHA Y10 phosphorylation in CRCSC spheres transfected with shRNAs and plasmids as indicated. (**b**,**c**) Lactate production levels and LDHA enzyme activity were determined in CRCSC spheres as indicated in **a**. (**d**) ECAR analysis of CRCSCs in **a**. (**e**) ECAR levels of CRCSCs treated with glucose (10 mM). (**f**) OCR analysis of CRCSCs in **a**. (**g**) OCR (basal respiration) levels of CRCSCs in **a**. (**h**) Western blot analysis of LDHA Y10 phosphorylation levels in colon organoid, CRCNSCs (CD133-negative CRC cells derived from patient) and CRCSCs (CD133-positive CRC cells) transfected with scrambled shRNA or hCINAP shRNA, respectively. (**i**–**m**) ECAR, OCR, NAD^+^/NADH, ROS and apoptosis levels (quantified from Supplementary Fig. 5c) were measured from cells in **h**. Data in **b**,**c**,**e**,**g**,**i**–**m** are are determined by Student's t-test and presented as mean±s.e.m. (*n*=3). NS, not significant, *P<0.05, **P<0.01, ***P<0.001. The unit of ROS (a.u.) is short for arbitrary unit.

LDHA reinforces the Warburg effect, providing colorectal tumour cells with a metabolic advantage that increases their chance of survival while facilitating invasion. Therefore, we investigated whether hCINAP acts as an up-regulator in the Warburg effect. hCINAP depletion attenuated LDHA Y10 phosphorylation in CRCSC spheres, and LDHA Y10 phosphorylation was recovered by re-expressing wild-type hCINAP, but not H79G mutant (Fig. 6a). Re-expressing wild-type hCINAP but not H79G mutant in CRCSC spheres with depleted hCINAP increased lactate production and LDHA activity (Fig. 6b,c). Furthermore, hCINAP depletion inhibited glycolysis and elevated OXPHOS in CRCSCs, which was rescued by wild-type hCINAP, but not by H79G mutant (Fig. 6d–g). In addition, treatment with rotenone, a mitochondrial complex I inhibitor, accompanying glycolysis inhibition induced by hCINAP shRNA, resulted in further decrements in cellular ATP level and cell viability (Supplementary Fig. 5h,i). These results indicate that hCINAP enhances the Warburg effect and restrains mitochondrial OXPHOS in CRCSCs relying on its adenylate kinase activity.

Generally, pyruvate from glycolysis fluxes into two routes: one is to produce lactate in cytosol with concomitant increased NAD^+^/NADH level, which is catalysed by LDHA, or to generate acetyl-CoA in mitochondiral citric acid cycle, elevating oxygen consumption and ROS level. Some cancer cells including CSCs tend to promote lactate generation and inhibit OXPHOS and ROS production for survival^42^. Therefore, we examined the functions of hCINAP in regulating the metabolism, ROS level and cell death of normal colon stem cell using organoid by isolating colon crypts from the adjacent tissue of colon cancer patients (Supplementary Fig. 5b), CRCNSCs (colorectal cancer non-stem cells, CD133-negative CRC cells selected by FACS) and CRCSCs (CD133-positive CRC cells). We found that LDHA Y10 phosphorylation or hCINAP level exhibited no significant difference among organoid, CRCNSCs and CRCSCs (Fig. 6h). While CRCNSCs showed lower glycolysis levels and higher oxygen consumption rate than those in CRCSCs and colon stem cells (Fig. 6i,j). However, hCINAP depletion led to larger decrease of ECAR and increases of OCR and NADH/NAD^+^ ratio in CRCSCs than those in CRCNSCs (Fig. 6i–k). And it has been reported that OCR level reflects OXPHOS and cellular ROS level, and high NADH/NAD^+^ ratio stimulates ROS production by alpha-ketoglutarate dehydrogenase^43^. Indeed, hCINAP knockdown caused more ROS production and higher apoptosis rate in CRCSCs than in CRCNSCs (Fig. 6l,m; Supplementary Fig. 5c,f,g). While colon organoid exhibited low sensitivity to hCINAP knockdown-induced changes in ECAR, OCR, NADH/NAD^+^ ratio, ROS level and apoptosis rate (Fig. 6i–m; Supplementary Fig. 5c,f,g). And we found that hCINAP depletion lowered cell viability but not affected differentiation of CRCSCs and colon organoid (Supplementary Fig. 5d,e). These data indicate that adenylate kinase hCINAP is requisite for CRCSC vitality by maintaining glycolytic and relatively low-ROS state.

**Figure 7:**
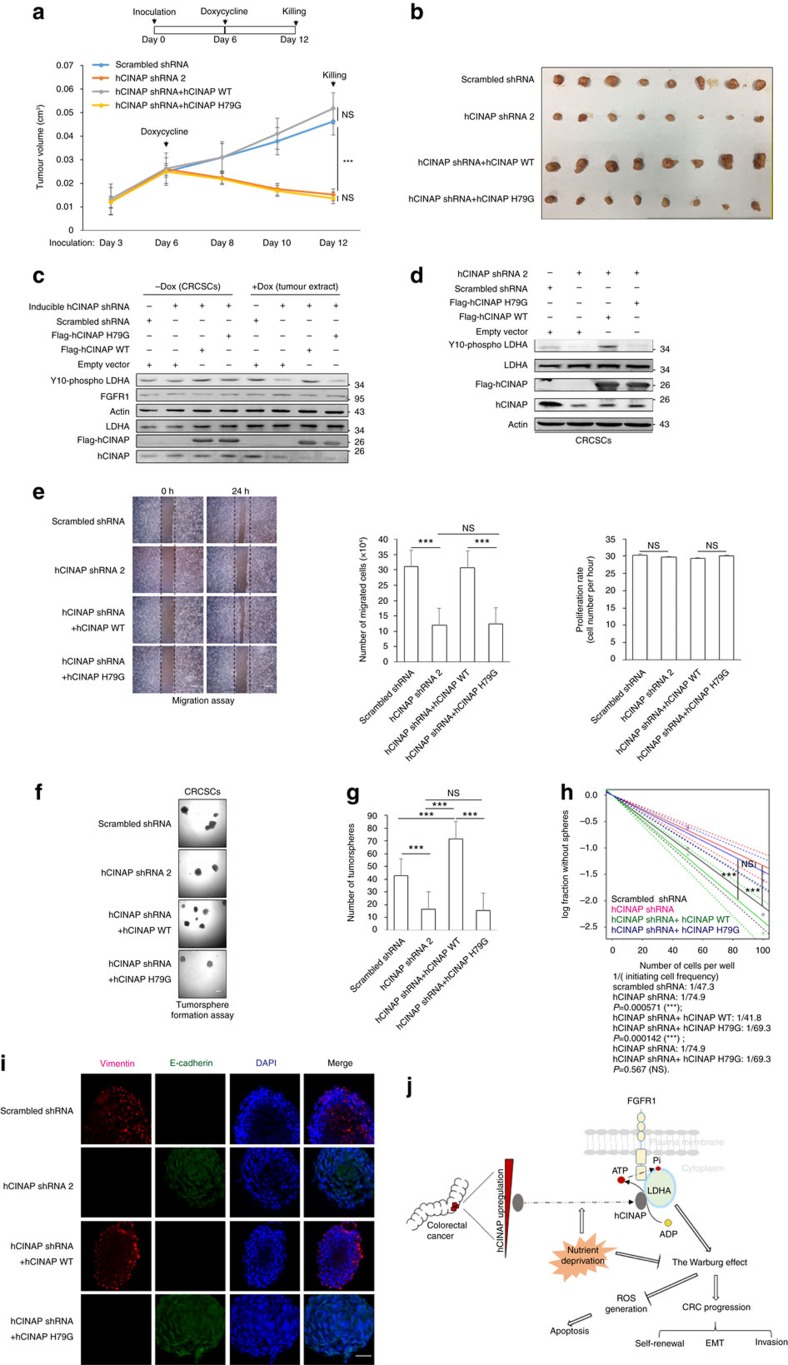
hCINAP depends on its adenylate kinase activity to promote CRCs growth and invasion. (**a**,**b**) Nude mice were subjected to orthotopic injection with CRCSCs expressing shRNAs and vectors as indicated and administrated with doxycycline 6 days later. The mice were then sacrificed 6 days later, after which tumours were collected, followed by photography and measurement. The tumour volumes from mice were quantified by paired *t*-test (*n*=8), NS, not significant, ***P<0.001. (**c**) Western blot analysis of LDHA Y10 phosphoryaltion in CRCSCs (without doxycycline administration) and tumour extract from xenograft mice (with doxycycline treatment). (**d**) LDHA Y10 phosphorylation levels in CRCSCs transfected as indicated in western blot. (**e**) Migration assay of SW480 cells and quantification of cells migration potential and proliferation rate. (**f**,**g**) Tumorsphere formation assay for CRCSCs in **d** and quantification by *t*-test (*n*=3), NS, not significant, ***P<0.001. (**h**) Limiting dilution assay for CRCSCs in **d**. Tumour-initiating frequency was calculated by extreme limiting dilution analysis (NS, not significant, ***P<0.001). (**i**) Immunofluorescence assay for the effect of hCINAP knockdown by shRNA and hCINAP wild-type or H79G mutant rescue expression on the mesenchymal–epithelial transition in CRCSC spheres. Vimentin (red), E-cadherin (green) and the nucleus (DAPI, blue). Scale bar, 100 μm. (**j**) A schematic working model to illustrate that hCINAP promotes the Warburg effect to maintain CRCSCs survival and self-renewal through its adenylate kinase activity-dependent upregulation of LDHA Y10 phosphorylation.

Next, we tested whether the adenylate kinase activity of hCINAP was essential for CRC growth and invasion. In orthotropic xenografted nude mice, hCINAP depletion induced by doxycycline administration suppressed LDHA Y10 phosphorylation (Fig. 7c) and significantly impaired tumour growth *in vivo*, whereas re-expression of wild-type hCINAP, but not H79G mutant hCINAP, in hCINAP-depleted CRCSCs compensated for the knockdown effect on tumour growth. We also found that hCINAP depletion had a rapid effect on tumour volume augment in hCINAP knockdown and hCINAP H79G mutant rescue groups within two days (days 6–8). However, in control and hCINAP wild-type rescue groups, tumour growth was not significantly affected. Moreover, sustaining doxycycline treatment resulted in further decrement on tumour volume in hCINAP knockdown and hCINAP H79G mutant rescue groups at days 10 and 12, and had no effect on tumour growth in shRNA control and hCINAP wild-type rescue groups. These results indicate that hCINAP depletion inhibits LDHA Y10 phosphorylation in CRCSCs and induces tumour bulk degeneration *in vivo* by diminishing the colorectal cancer stem cell pool, instead of delaying tumour cells growth (Fig. 7a,b). Excessive lactate produced in the process of aerobic glycolysis is mostly secreted into the extracellular matrix, remodelling the CRCSCs niche and conferring tumour cells with stem cell-like properties and migration capacity^35^. Migration assays were performed to evaluate the stemness and invasive capacity of CRCSCs. As expected, hCINAP depletion impeded the migratory capacity of SW480 cells; wild-type hCINAP restored cell invasiveness, but H79G mutant hCINAP did not (Fig. 7e). Consistently, similar results were obtained in tumorsphere formation, limiting dilution assays and EMT program assessed by immunofluorescence assays (Fig. 7d,f–i). These results indicate that hCINAP, dependently of its adenylate kinase activity, promotes CRCSCs growth and stemness maintenance by enhancing the Warburg effect.

Finally, we investigated the clinical significance of the relationship between hCINAP and LDHA Y10 phosphorylation. Consistently, expression levels of Y10-phosphorylated LDHA and hCINAP were higher in tumour tissue from fifty CRC patients in comparison with the levels in adjacent tissues (Supplementary Fig. 1a,b). Moreover, hCINAP abundance was positively correlated with the level of Y10-phosphorylated LDHA normalized to the total LDHA protein level (Supplementary Fig. 1b).

Overall, our studies demostrate that hCINAP drives colorectal tumour growth and invasion via its adenylate kinase activity by enhancing LDHA Y10 phosphorylation (Fig. 7j), and provides a potential therapeutic solution in the treatment of colorectal cancer.

## Discussion

The Warburg effect implies that cancer is a set of metabolic diseases^17^. Cancer cells tend to produce ATP via glycolysis followed by lactate production, instead of mitochondrial OXPHOS, even under normoxic conditions. Metabolites are diverted to anabolism to provide cellular building blocks for the biosynthetic requirements of rapid proliferation in multiple cancers. Likewise, proliferating pluripotent stem cells, including iPSCs, are also predominantly glycolytic^44^. The various metabolic phenotypes of CSCs are dependent upon cancer type and sometimes heterogeneous^45^. CSCs in pancreatic cancer^45^, glioblastoma and glioma^46^,^47^, and leukaemia^48^,^49^ are dependent on OXPHOS, whereas CSCs in breast cancer^42^,^50^ and nasopharyngeal cancer^51^ are mainly glycolytic. However, the metabolic phenotype of CRCSCs has not been studied comprehensively. Our results suggest that CRCSCs obtain energy predominantly by glycolysis. Compared to CRCNSCs (CRC non-stem cells), CRCSCs show higher glycolysis levels. Inhibition of CRCSC LDHA activity by hCINAP RNAi depletion markedly attenuated the extracellular acidification rate (ECAR), self-renewal, resistance to conventional chemotherapeutics, and tumour formation *in vivo* and *in vitro*, indicating that the Warburg effect is beneficial for CRCSCs because it confers a metabolic advantage and facilitates adaption in an adverse environment. Interestingly, it was reported that a subpopulation of CD133^+^ CRCSCs isolated from primary tumours had a level of glycolysis significantly higher than that of CD133^−^ cells^31^, consistent with the metabolic traits of CRCSCs in our research.

It is worth mentioning that hCINAP inhibition resulted in significant cell death in both CRCNSCs and CRCSCs by shunting from glycolysis to OXPHOS and aggravating ROS generation. Moreover, although normal colon stem cells as well as CRCSCs showed high glycolysis level, hCINAP depletion caused slight metabolic reprogramming and had no remarkable impact on apoptosis in colon organoid, suggesting that normal colon stem cells possess greater metabolic plasticity and resistance to apoptosis than CRCSCs. On the basis of this, there would be a potent therapeutic efficacy and a low gut toxicity by targeting hCINAP expression and activity in CRC.

The EMT is a conserved program in the development of multicellular organisms, in which stationary epithelial cells obtain migratory and invasive ability and mesenchymal properties^52^. In CSCs, many intracellular signalling pathways, including the TGF-β, Wnt/β-catenin, Notch and NF-κB pathways^53^, are activated by the CSC microenvironment constituents, including cytokines, chemokines, growth factors and cancer-associated fibroblast^54^, upon which they transduce signals to transcriptional regulators, including SNAIL, TWIST and ZEB, and EMT effectors such as E-cadherin and vimentin^55^. Here we report that hCINAP depletion reversed the mesenchymal state of CRCSCs, reducing expression of TWIST1, SNAIL1, fibronectin and vimentin and upregulating E-cadherin and γ-catenin. Moreover, CRCSC markers, including CD44 and LGR5, are the target genes of the Wnt pathway, indicating that CSCs take advantage of the EMT to sustain self-renewal and maintain invasiveness. Indeed, we found that hCINAP depletion also reduced the CD133 and LGR5 levels of CRCSCs. Collectively, these results suggest that hCINAP determines the signature of CRCSCs via the EMT.

Mechanistically, hCINAP depends on its adenylate kinase activity to facilitate Y10 phosphorylation of LDHA by FGFR1. Protein kinases transfer phosphate groups from ATP to proteins, whereas adenylate kinases catalyse the conversion of two molecules of ADP into one molecule of ATP and one molecule of AMP, thereby acting as key modulators of cellular energy homeostasis^40^. Strikingly, this study initially indicated that protein phosphorylation was facilitated by the enzymatic activity of an adenylate kinase. AK2 activity is associated with mitochondrial energy metabolism and the unfolded protein response in humans^56^; moreover, it positively regulates FADD phosphorylation and cell growth^57^. Rad50 adenylate kinase activity is involved in repairing DNA double-strand breaks by forming Mre11/Rad50 complexes^58^. However, how the activity of adenylate kinase functions in these pathways remained to be answered. Adenylate kinases consist of three well-established domains: the rigid CORE domain, an ATP binding LID domain, and an AMP binding domain. The conformational transition involved in conversion of ATP and AMP to ADP have been proposed^59^, while the inverse process remains to be elucidated. In this study, we showed that energy shortage induced interaction between LDHA and hCINAP, thus reinforcing the resistance of CRCSCs to adverse nutrient conditions, and we wondered whether cellular AMP/ATP ratio fluctuation caused by nutrient deprivation determines the conformation of hCINAP and thus influences LDHA–hCINAP interaction, reminiscent of the best-known energy sensor, AMP-activated protein kinase (AMPK). Interestingly, if we regard hCINAP as a regulatory subunit of LDHA, activation of the LDHA–hCINAP complex has striking similarities with that of AMPK. AMPK activation relies on phosphorylation at T172 of the catalytic α-subunit by CaMKKβ or LKB1, and AMP binding to the AMPK γ-subunit promotes AMPK α-subunit T172 phosphorylation^60^. Increasing the AMP/ATP ratio by nutrient deprivation facilitates both AMPK and LDHA phosphorylation; however, AMP promotes AMPK phosphorylation by inhibiting PP2C-directed α-T172 dephosphorylation^61^, whereas AMP stimulates LDHA phosphorylation by enhancing LDHA–hCINAP interaction. Further study is required to elucidate the crosstalk between LDHA and AMPK signalling.

Phosphorylation enhances the catalytic activity of LDHA, aerobic glycolysis, and production of lactate, which is secreted by lactate transporters known as monocarboxylate transporters. Monocarboxylate transporters transport lactate out of cells in a process dependent on the lactate concentration gradient and coupled with proton secretion, thereby causing extracellular acidification^62^. Notably, lactate alters the cancer microenvironment by inducing secretion of hyaluronan by cancer-associated fibroblasts, which is beneficial for cancer cell migration and invasion^35^,^63^; moreover, hyaluronan inhibits immune surveillance by monocytes and T cells^64^,^65^,^66^,^67^, as well as cytokine release^68^. In addition, lactate levels in cancer patients can be used to predict metastasis and recurrence^69^.

Taken together, the results of this study demonstrate that hCINAP positively regulates LDHA activity, thus enhancing aerobic glycolysis and CRC progression via tripartite mechanisms (Fig. 7j): (1) LDHA promotes biosynthesis to support CRC cell proliferation; (2) LDHA generates adequate extracellular lactate to provide a favourable microenvironment for CRCSC growth and invasion; and (3) hCINAP promotes CRCSCs to be more glycolytic and less OXPHOS-dependent to astrict cellular ROS overproduction and maintain survival. Thus, our study reveals that the metabolic features of CRCSCs are tightly regulated by oncogenic adenylate kinase hCINAP. Targeting both CRCSCs and non-stem CRC cells is desirable because non-stem cancer cells within CRC tumours can dedifferentiate into CRCSCs^33^; simultaneous targeting of CRCSCs and non-stem CRC cells can be achieved using anti-glycolytic drugs. Future studies should assess the effects of small-molecule hCINAP inhibitors and monoclonal antibodies targeting hCINAP on CRC cells.

## Methods

### Establishment of CRCSC cultures

Human colorectal cancer samples were obtained from Peking University Third Hospital. All patients signed an informed consent in accordance with the ethical standards of the Peking University Third Hospital Ethical Committee. Fresh resected CRC tissues were mechanically and enzymatically dissociated into single-cell suspensions and cultured in CRCSC culture medium (available in the Cell culture and transfection). The studies were performed after approval by the Ethics Committee of Peking University Caner Hospital and Institute. FACS was used to sort for CD133 and LGR5 double-positive cells by using CD133-PE and LGR5-APC microbeads (Miltenyi Biotec). Sorted cells were cultured until tumorspheres formed. Tumorspheres were dissociated into single cells, plated to adhere in serum-containing and heparin-free DMEM, and differentiated into sphere-derived adherent cells.

### Tumorsphere formation assay

Cells were plated into 6-well ultra-low attachment plates (Corning) at a density of 20,000 cells per ml and cultured in CRCSC culture medium. Tumorspheres were cultured for 12 days, collected by centrifugation (600 r.p.m.), fractionated using 100-μm cell strainers (Corning), and counted by a light microscope.

### Transwell invasion assay

Cells were suspended at a density of 40,000 cells per ml in CRCSC medium and seeded into matrigel-covered (Corning) 8 μm sieves on 12-well plates (1 ml per plate) coated with 1.2% polyhema. After 2 days, cells on the upper chamber were removed, whereas cells on the lower surface of the sieve were fixed and stained with crystal violet. Cells that migrated through the sieve were observed and counted under a light microscope.

### Measurement of ECARs and OCRs

ECARs and OCRs were analysed using an XF24 extracellular flux analyzer (Seahorse Bioscience) following the manufacturer’s protocol.

### Limiting dilution assay

Single cells dissociated from colorectal tumorspheres were seeded in 96-well plates containing CRCSC culture medium (50 or 100 cells per well). Ten days later, tumorsphere formation was examined in each well. CRCSC frequency was calculated by extreme limiting dilution analysis following the procedure described in http://bioinf.wehi.edu.au/software/elda.

### Nude mice xenograft assay

CRCSCs (1 × 10^6^) stably transfected with PLKO Puro hCINAP or control RNAi lentivirus and rescued by Flag-hCINAP wild type or H79G were injected into the left and right flanks of BALB/c nude mice (7 weeks of age, female, 16 mice were divided into two groups randomly). Mice drinking water containing doxycycline (0.5 mg ml^−1^) was administrated 6 days after inoculation of cells. Tumour size was monitored three times per week. Mice were sacrificed 6 days after doxycycline administration, upon which tumours were isolated and measured. The nude mice tumorigenesis assay was approved by the Peking University Laboratory Animal Center. All animals were handled following the ‘Guide for the Care and Use of Laboratory Animals’ and the ‘Principles for the Utilization and Care of Vertebrate Animals’.

### Plasmids and reagents

The cDNAs of human hCINAP (wild-type and H79G mutant), the FGFR1 intracellular kinase domain containing residues 1–310, and LDHA were amplified by PCR and cloned into the pcDNA3.0-3flag, pRK-HA, pGEX4T1 and pET28a vectors. The following antibodies were used in this study: mouse monoclonal Flag antibody (F3165, Sigma, USA; 1:2,000), HA antibody (H9658, Sigma, USA; 1:2,000), mouse monoclonal His antibody (D291-3, MBL, Nagoya, Japan; 1:2,000), mouse monoclonal Myc antibody (M047-3, MBL, Nagoya, Japan; 1:1,000), mouse monoclonal GST antibody (M071-3, MBL, Nagoya, Japan; 1:1,000), rabbit monoclonal ACTIN antibody (PM053, MBL, Nagoya, Japan; 1:2,000), rabbit polyclonal LDHA antibody (BS6179, Bioworld Technology Inc.; 1:2,000), rabbit polyclonal hCINAP antibody (BS2171, Bioworld Technology Inc.; 1:1,000), rabbit polyclonal CD44 antibody (A1351, Abclonal; 1:1,000), rabbit polyclonal CD133 antibody (A0219, Abclonal; 1:1,000), rabbit polyclonal LGR5 antibody (A8090, Abclonal; 1:1,000), rabbit polyclonal HIF1 alpha antibody (GTX127309, GeneTex; 1:500), rabbit polyclonal FGFR1 antibody (I648, Bioworld Technology, Inc.; 1:500) and Goat polyclonal LGR5 antibody (sc-68,580, Santa Cruz Biotechnology; 1:50 for immunofluorescence). Polyclonal phospho-LDHA antibody (Tyr10) (#8,176; 1:500), rabbit polyclonal c-Abl antibody (#2,862; 1:1,000), rabbit polyclonal FGFR1 antibody (#9,740; 1:1,000), mouse monoclonal phospho-FGF receptor (Tyr653/654) antibody (#3,476; 1:500), mouse monoclonal JAK2 antibody (D21E2; 1:1,000), rabbit monoclonal E-cadherin antibody (#14,472; 1:50 for immunofluorescence) and mouse monoclonal vimentin antibody (#D32H3; 1:100 for immunofluorescence) were purchased from Cell Signaling Technology (USA). IgG2b-FITC isotype control (Cat No. 130-098-415), IgG2b-APC isotype control (Cat No. 130-098-414), CD133-FITC (Cat No. 130-105-226) and LGR5-APC (Cat No. 130-100-854) were from Miltenyi Biotec (1:50 for FACS). AP5A (D8013) was purchased from Sigma (USA). NVP-BGJ398 (S2183) was from Selleck.cn (Shanghai). TRIzol Reagent was purchased from cwbiotech (CW0580).

### Cell culture and transfection

SW480 cells were purchased from American Type Culture Collection (ATCC, CCL-228) and cultured in DMEM (Gibco) with 10% fetal bovine serum (HyClone) at 37 °C in a 5% CO_2_ incubator. Patient-derived CRC cells were cultured in CRCSC culture medium containing DMEM/F12 supplemented with heparin (2.5 μg ml^−1^), B27 (Gibco), N2 (Gibco), basic fibroblast growth factor (bFGF; 20 ng ml^−1^) and epidermal growth factor (EGF; 20 ng ml^−1^) in a humidified 5% CO_2_ incubator at 37 °C. CRCSC tumour-initiating spheres were characterized using CD133 (ABClonal, Wuhan), LGR5 (ABClonal, Wuhan) and CD44 (ABClonal, Wuhan) stem cell markers. All cells were transfected with plasmids using PEI (Polyscience, USA) following the manufacturer’s instructions and under Plasmocin (25 μg ml^−1^, Invivogen, USA) treatment.

### Wound-healing assay

For the wound-healing assay, monolayer cells were wounded with a sterile plastic tip. Cell migration was observed by microscopy after 24 h and quantified using ImageJ software according to the manufacturer’s instructions.

### Immunoprecipitation and immunoblotting

Cells were trypsinized, collected and lysed for 2 h in cell lysis buffer containing 0.3% Nonidet P-40, 50 mM Tris-HCl (pH 7.5), 150 mM NaCl, 1 μg ml^−1^ aprotinin, 1 μg ml^−1^ leupeptin, 1 μg ml^−1^ pepstatin, 1 mM Na_3_VO_4_ and 1 mM PMSF. Cell lysates were pre-cleaned with 30 μl protein G, after which 500 μl of each cell lysate was incubated with the indicated antibody or IgG at 4 °C for 4 h. Next, protein G was added to the lysate, which was incubated at 4 °C for 4 h. The beads were washed three times with NP40 cell lysis buffer. Whole-cell lysates (10%) were mixed with loading buffer and heated at 96 °C for 10 min. Samples were fractionated by SDS–PAGE and transferred to nitrocellulose membranes (GE Healthcare, China). The membranes were blocked in 5% milk in PBS (5% BSA in PBS for blocking of Y10 phosphorylated-LDHA) at room temperature for 1 h and incubated in primary antibodies for 2 h, after which they were incubated with secondary antibodies for 1 h at room temperature. Fluorescence signals on nitrocellulose membranes were detected and analysed using an Odyssey Infrared Imaging System (LI-COR Biotechnology). Uncropped scans of all blots are shown in Supplementary Fig. 8.

### RNA interference-mediated knockdown and qRT–PCR

hCINAP knockdown was carried out using lentiviruses containing shRNA oligonucleotides: hCINAP shRNA 1, 5′-CAGAGUAGUUGAUGAGUUA-3′; and hCINAP shRNA 2, 5′-GAGAGAAGGUGGAGUUAUU-3′. A scrambled shRNA lentivirus with no effect on hCINAP was constructed as a negative control using the following sequence: 5′-UUCUCCGAACGUGUCACGU-3′ (GeneChem Co. Ltd. China). LDHA was depleted by using shRNA oligonucleotide: 5′-GGAGAAAGCCGUCUUAAUU-3′, which was constructed in PLKO.1 puro vector (Addgene) and then packaged into lentivirus system with pMD2.G and pSPAX2 vectors. The RNAi lentiviruses were transfected into cells using polybrene (8 μg ml^−1^) to enhance infection efficiency. Puromycin (2 μg ml^−1^) was added to the cells for 72 h to screen for stably infected cells. Total RNA from the cells was extracted with TRIzol reagent following the manufacturer’s instructions (Primers used in qRT–PCR were attached in Supplementary Table 1).

### *In vitro* pull-down assay

Recombinant glutathione S-transferase-tagged hCINAP, hCINAP (H79G), LDHA and FGFR1 1–310 truncated fragments, as well as His-tagged LDHA and hCINAP proteins, were expressed and purified from *E. coli* BL21(DE3). GST pull-down assays were performed by incubating GST-tagged recombinant proteins immobilized on glutathione-sepharose resin (BD Biosciences, San Jose, CA, USA) with His-tagged proteins at 4 °C for 3 h. Similarly, His pull-down assays were performed by incubating nickel resin-associated recombinant His-tagged proteins with GST-tagged proteins at 4 °C for 3 h. Beads were washed to remove non-specific contaminants, after which specific binding proteins were eluted and subjected to SDS–PAGE analysis.

### *In vitro* kinase assay

In brief, His-tagged LDHA purified from *E. coli* was incubated with active recombinant GST-tagged FGFR1 at room temperature for 30 min in FGFR1 kinase buffer containing 150 mM NaCl, 5 mM DTT, 10 mM MnCl_2_, 0.01% Triton X-100, 10 mM Hepes (pH 7.5) and 200 mM ATP. The samples were fractionated via 15% SDS–PAGE and immunoblotted with antibodies against phosphorylated LDHA Y10.

### LDHA enzyme activity assay and lactate production assay

LDHA protein from SW480 cells was immunoprecipitated and added to a reaction buffer containing 0.2 M Tris-HCl (pH 7.3), 0.05% bovine serum albumin, 10 mM MgCl_2_, 2 mM pyruvate and 20 μM NADH. The reduction in absorbance at 340 nm resulting from NADH oxidation was measured using a Tecan spectrophotometer. Lactate production was measured by detecting the decrease in fluorescence at 340 nm from theoxidation of cellular lactate with a lactate assay kit (Jian Cheng, Nanjing, China). Phenol red-free RPMI medium without serum was added to a six-well plate of subconfluent cells, which were incubated at 37 °C for 1 h. Next, 2 μl of medium from each well was assessed. Cell numbers were counted using a light microscope.

### Immunofluorescence

Colorectal tumorspheres were cytospun upon glass slides coated with poly-lysine. Sphere-derived adherent cells were plated on matrigel-coated cover slips. Cells were fixed in 4% paraformaldehyde, permeabilized in 0.1% Triton X-100 and blocked using PBS containing 1% BSA. And then cells were incubated with primary antibodies overnight at 4 °C, followed by incubation with FITC/TRITC-conjugated secondary antibodies at room temperature in the dark for 1 h and staining for 10 min with DAPI (Wako, Japan). Images were captured using a two-photon confocal laser scanning microscope (ZeissLSM 710).

### Cell viability measurement

Cells were added 0.05 mg ml^−1^ MTS (3-(4,5-dimethylthiazol-2-yl)-5-(3-carboxymethoxyphenyl)-2-(4-sulfophenyl)-2H-tetrazolium, inner salt) and phenazine ethosulfate (CellTiter 96 AQueous One Solution reagent, Promega) at 37 °C for 3 h and then digested by trypsin into single cells. Then the absorbance at 492 nm of cell solutions was measured.

### Subcellular fractionation

SW480 cells (1 × 10^6^) were collected and washed using ice-cooled PBS at 4 °C. Five per cent of cells was used as the whole-cell lysate fraction, and the rest cells were resuspended in hypotonic buffer (10 mM HEPES, pH 7.4, 10 mM KCl, 1.5 mM MgCl_2_, 0.5 mM DTT) with protease inhibitor cocktail for 10 min at 4 °C. The swollen cells were transferred to a pre-cooled Dounce homogenizer, homogenized for 15 times on ice, centrifuged at 1,000 r.p.m. for 5 min at 4 °C and resuspended with 250 μl Cytoplasmic Extract Buffer (0.3 M HEPES, pH 7.9, 1.4 M KCl, 0.03 M MgCl_2_, 0.5 mM DTT). The supernatant was collected as the cytosolic fraction, and the pellet was used as the nucleoplasm fraction.

### NADH/NAD^+^ ratio and ROS level measurement

The SoNar (sensor of NAD(H) redox) system was used for NADH/NAD^+^ ratio detection^70^. Briefly, cells were transfected with SoNar and cpYFP (control) plasmids and selected in 400 μg ml^−1^ hygromycin (Amresco) for 10 days. The fluorescence intensities excitated at 420 and 485 nm (normalized to cpYFP) of the stable cells were measured at 530 nm by the Microplate Reader (BioTek). Cells were added to 10 mM DCFH-DA (2,7-Dichlorodihydrofluorescein diacetate, Sigma) and incubated for 30 min at 37 °C. The fluorescence intensities (excitated at 420 nm and emitted at 485 nm) of the cells were measured by the Microplate Reader (BioTek).

### Fluorescence-activated cell sorting

Cells were trypinized, neutralized by complete media (DMEM/10% FBS). Cells were rinsed carefully to break up aggregates and centrifuged at 1,000 r.p.m. for 5 min. Discard the supernatant, resuspend the cells in PBS (1% BSA), and then add anti-CD133 (FITC), anti LGR5 (APC) and isotype control antibodies (for apoptosis assays, Alexa Fluor 488 conjugated Annexin V and Propidium iodide were added) into cells for at 37 °C for 10 min. Cells were centrifuged at 1,000 r.p.m. for 5 min and analysed by FACSVerse (BD bioscience).

### Colon organoid culture

Fresh resected human colon tissue, acquired with informed consent from all patients, in accordance with the ethical standards of the Peking University Third Hospital Ethical Committee and the Ethics Committee of Peking University, was washed by using PBS containing penicillin–streptomycin and cut up carefully to 25 mm^2^. Tissues were washed by PSB and digested by chelation buffer (5.6 mM Na_2_HPO_4_, 8.0 mM KH_2_PO_4_, 96.2 mM NaCl, 1.6 mM KCl, 43.4 mM sucrose, 54.9 mM D-sorbitol, 0.5 mM DL-dithiothreitol in distilled water) at 4 °C for 1 h. The supernatant was decanted carefully, chalation buffer was add and shaked vigorously to obtain supernatant containing colon crypts. The crypts were collected and mixed with DMEM, and then the mixture was transferred to preheated 96-well plates with concretionary 5 μl Matrigel (8 mg ml^−1^, BD bioscience). 15 min later, 100 μl colon organoid culture medium was supplied (Advanced DMEM/F12 (Invitrogen), B27 and N2 supplement (Invitrogen), 1 mM *N*-Acetyl-L-cysteine (Sigma-Aldrich), 10 mM GlutaMAX Supplement (Invitrogen), 10 mM HEPES (Invitrogen), Primocin (Invitrogen), 1,000 ng ml^−1^ Respondin-1 (Sino biological inc.), 100 ng ml^−1^ m-noggin (Peprotech), 50 ng ml^−1^ m-EGF (Invitrogen), 10 nM [Leu15]-Gastrin I (Sigma-Aldrich), 500 nM A-83-01 (Tocris), 10 μM SB202190 (Sigma-Aldrich), 100 ng ml^−1^ mouse recombinant Wnt-3A (GTX109037, GeneTex), 10 mM Nicotinamide (Sigma-Aldrich)) for the colon crypts at cell incubator.

### Immunohistochemistry analyses

Human colorectal adenocarcinoma tissue arrays were from Shanghai Biochip Company Ltd (Shanghai, China) and stained by using rabbit anti-human hCINAP (1:4,000) as the primary antibody and then incubated with 3, 30-diaminobenzidine and a negative control sample was treated identically (without the primary antibody). A Leica DM IRE2 microscope (Leica Microsystems Imaging Solutions Ltd, Cambridge, UK) was used for the imaging and measurement of the hCINAP staining intensity.

### Bioinformatic analysis of hCINAP expression in CRC

hCINAP expression was analysed in CRC based on the TCGA COAD (Colon adenocarcinoma) and READ (Rectum adenocarcinoma) cancer types. For analysis of hCINAP differential hCINAP expreesion in colorectal cancer or normal tissue, we applied one-way analysis of variance (ANOVA), using disease state (Tumour or Normal) as the variable for calculating differential expression. The expression data for differential analysis were log_2_(TPM+1) transformed (TPM: transcripts per million). And we defined the differentially expressed genes as those satisfying following thresholds: *P*(disease state)<0.01. For analysis of hCINAP differential hCINAP expreesion in colorectal cancer four pathological stages, the differential hCINAP expression data were transformed by using log_2_(TPM+1). And for analysis of hCINAP differential expression in four consensus molecular subtypes (CMSs)^29^, we profiled the hCINAP expression in each subtype. And one-way ANOVA was applied for hypothesis testing.

### Statistical analysis

All results for continuous variables are presented as mean and s.e.m. (Student’s *t*-test, two-sided) unless otherwise stated. *P*-values <0.05 were considered significant (variance is similar between the groups). Analysis and graphical presentation were performed using Graphpad Prism 5.0 software and Microsoft Excel 2013.

### Data availability

The authors declare that all the relevant data (sequences and software code) in this study are available from Supplementary Information Files or the authors upon reasonable request. The hyperlink and doi for hCINAP expression in CRC data set are https://figshare.com/s/75c1b6f12bee15e81fb0 and doi: 10.6084/m9.figshare.4737181.

## Additional information

**How to cite this article:** Ji, Y. *et al*. Adenylate kinase hCINAP determines self-renewal of colorectal cancer stem cells by facilitating LDHA phosphorylation. *Nat. Commun.* **8,** 15308 doi: 10.1038/ncomms15308 (2017).

**Publisher’s note:** Springer Nature remains neutral with regard to jurisdictional claims in published maps and institutional affiliations.

## Supplementary Material

Supplementary Information Supplementary Figures and Supplementary Tables

Peer Review File
