# Corrigendum: Adenylate kinase hCINAP determines self-renewal of colorectal cancer stem cells by facilitating LDHA phosphorylation

**DOI:** 10.1038/ncomms16000

**Published:** 2017-06-16

**Authors:** Yapeng Ji, Chuanzhen Yang, Zefang Tang, Yongfeng Yang, Yonglu Tian, Hongwei Yao, Xi Zhu, Zemin Zhang, Jiafu Ji, Xiaofeng Zheng

Nature Communications 8: Article number:15308; DOI: 10.1038/ncomms15308 (2017); Published: 05 18 2017; Updated: 06 16 2017

The original version of this Article contained an error in the spelling of the author Zemin Zhang, which was incorrectly given as Zeming Zhang. This has now been corrected in both the PDF and HTML versions of the Article.

