# Supplementary material for: Adenylate kinase hCINAP determines self-renewal of colorectal cancer stem cells by facilitating LDHA phosphorylation
Source: Nat Commun. 2017 May 18;8:15308. doi: 10.1038/ncomms15308 (PMC5454382; doi:10.1038/ncomms15308)
Supplement: Supplementary Information — Supplementary Figures and Supplementary Tables [file ncomms15308-s1.pdf]

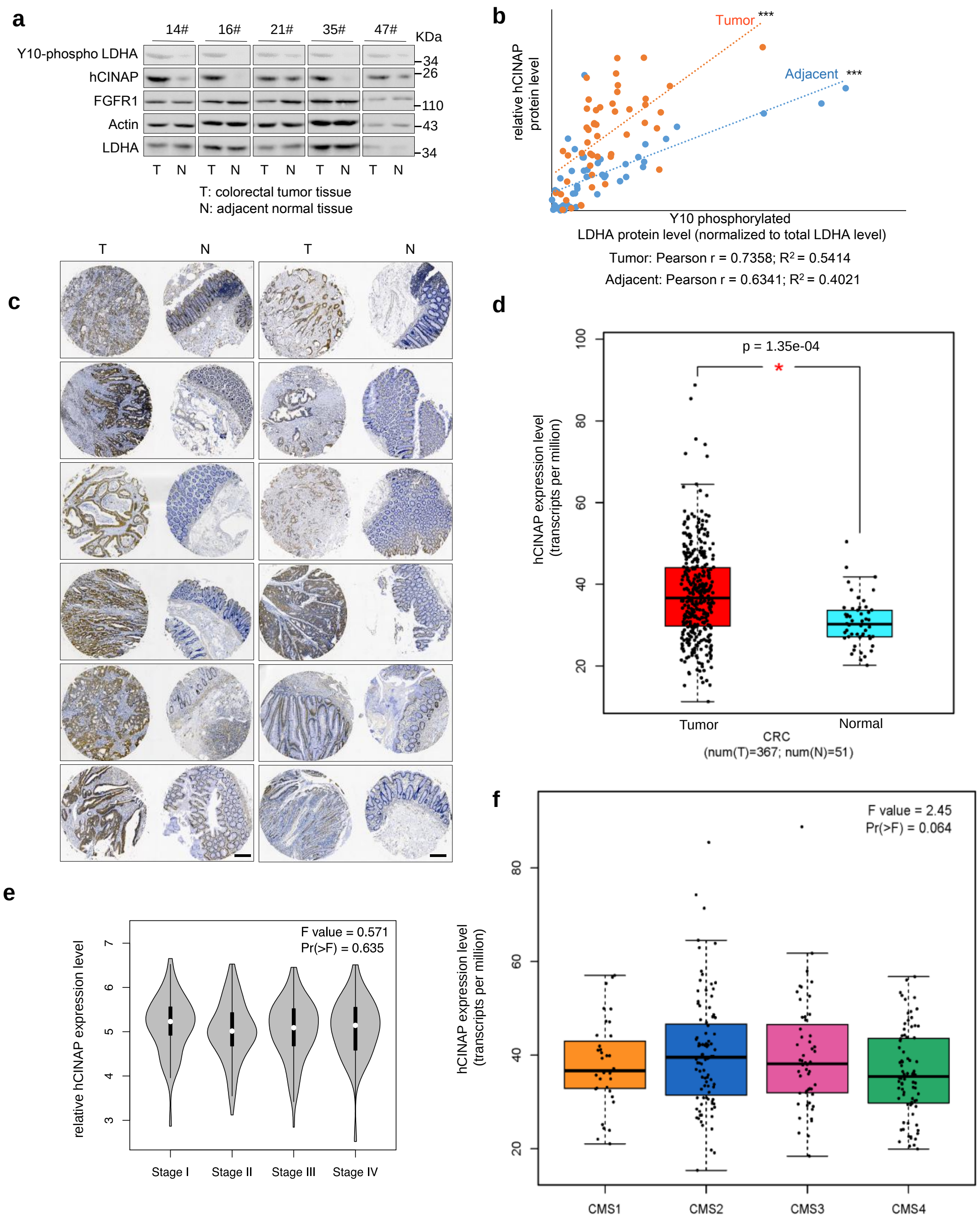

Supplementary Figure 1. hCINAP is highly expressed in colorectal cancer. (a) Western blot analysis of the levels of Y10-LDHA, hCINAP, LDHA and Actin in tissues from CRC patients. (**b**) Correlation analysis of Y10-phosphorylated LDHA and hCINAP in CRC tissues and adjacent normal tissues. Correlations were analyzed by Pearson test. Data are presented as mean ± SEM. NS, not

significant, ***$p<0.001$. (**c**) Representative immunohistochemical staining for hCINAP expression in CRC tissue microarrays. Scale bar, 200 μm. (**d**) Analysis of the hCINAP expression in CRC based on the TCGA COAD (Colon adenocarcinoma) and READ (Rectum adenocarcinoma) cancer types. Differential expression of hCINAP in tumor or normal tissue was calculated by one-way analysis of variance (ANOVA), $p<0.001$. (**e**) Violin-plots represent hCINAP expression in four stages of CRC by ANOVA. (**f**) Box-plots indicate hCINAP expression in four consensus molecular subtypes of CRC. The one-way ANOVA are applied for hypothesis testing.

a

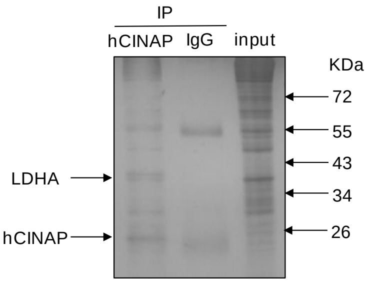

b

Reference	Peptide	Probability	Score
LDHA	K.DLADELALVDVIEDK.L	8.79E-12	30.18
nucleophosmin	K.DELHIVEAEAMNYEGSPIK.V	5.71E-13	70.29
PP1A	K.QSLETICLLLAYK.I	4.84E-09	30.27
ribosomal protein L6	R.HQEGEIFDTEKEKYEITEQR.K	1.82E-08	70.28

Supplementary Figure 2. Identification of hCINAP-binding partners using IP-MS. (**a**) Western blot and silver-staining analysis of immunoprecipitation assay in SW480 cells using endogenous hCINAP antibodies. Rabbit IgG served as the negative control. Arrows indicate immunoprecipitated hCINAP and candidate LDHA. (**b**) Identification of hCINAP interacting proteins via mass spectrometry analysis.

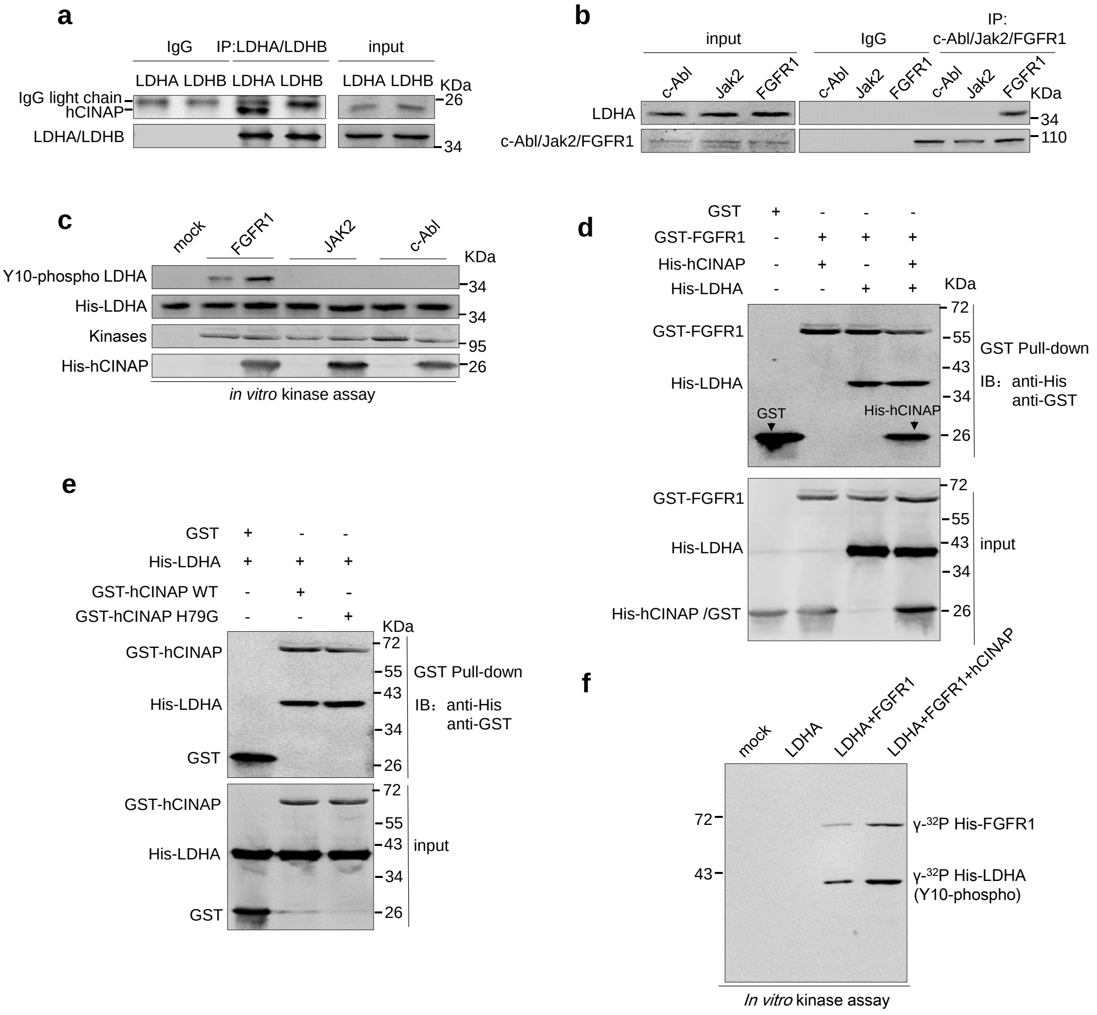

Supplementary Figure 3. FGFR1, LDHA and hCINAP form a ternary complex. (**a**) Co-IP analysis of the interaction between endogenous hCINAP with LDHA or LDHB in SW480 cells. (**b**) Co-IP identified FGFR1, but not c-Abl and JAK2, interacted with LDHA in SW480 cells. (**c**) *In vitro* LDHA phosphorylation assay by using His-LDHA and FGFR1/c-Abl/JAK2 kinases immunoprecipitated from SW480 cells, with or without His-hCINAP. (**d**) *In vitro* GST pull-down analysis of the interaction among His-LDHA, His-hCINAP and GST-FGFR1. (**e**) *In vitro* GST pull-down assay by using GST-hCINAP (wild-type and H79G mutant) and His-LDHA. (**f**) *In vitro* FGFR1 kinase assay by using γ-^{32}P labeled ATP, with or without His-hCINAP.

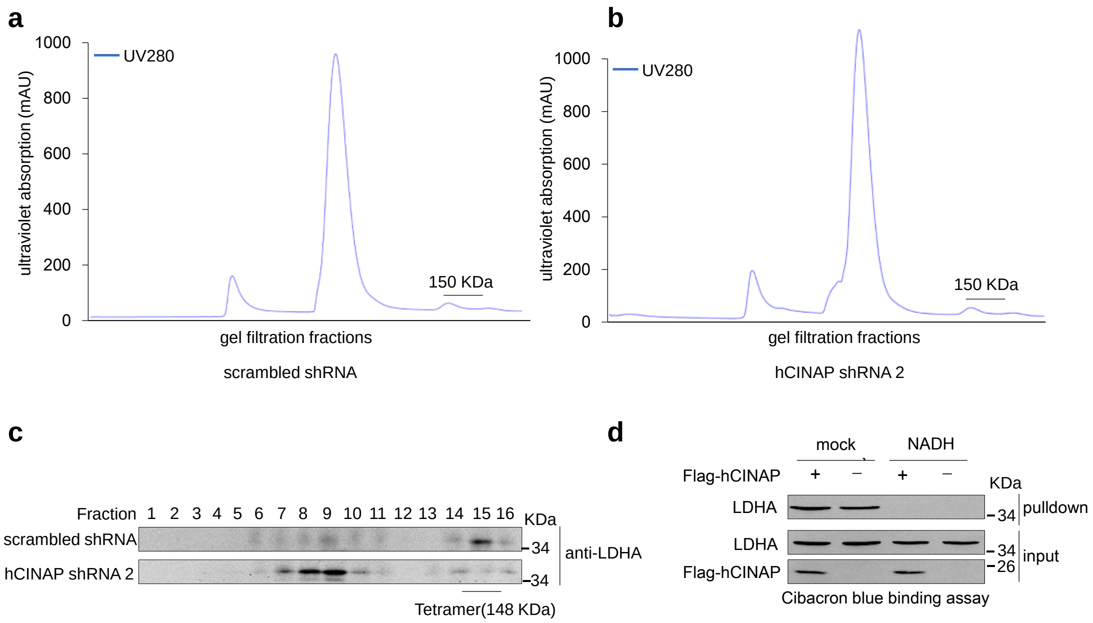

Supplementary Figure 4. LDHA tetramerization is enhanced by hCINAP. (**a**) and (**b**) Gel filtration analysis of whole lysates from 1×10^8 SW480 cells stably transfected with control shRNA or hCINAP shRNA. (**c**) Western blot analysis of LDHA protein level in various fractions collected via gel filtration. Molecular weights of eluted fractions separated by a Sephacryl S-200 HR column (GE Healthcare) were marked by ovalbumin (43 kDa), conalbumin (75 kDa) and aldolose (158 kDa). (**d**) NADH binding assay using NADH analog Cibacron blue 3GA, with or without NADH as a competitor.

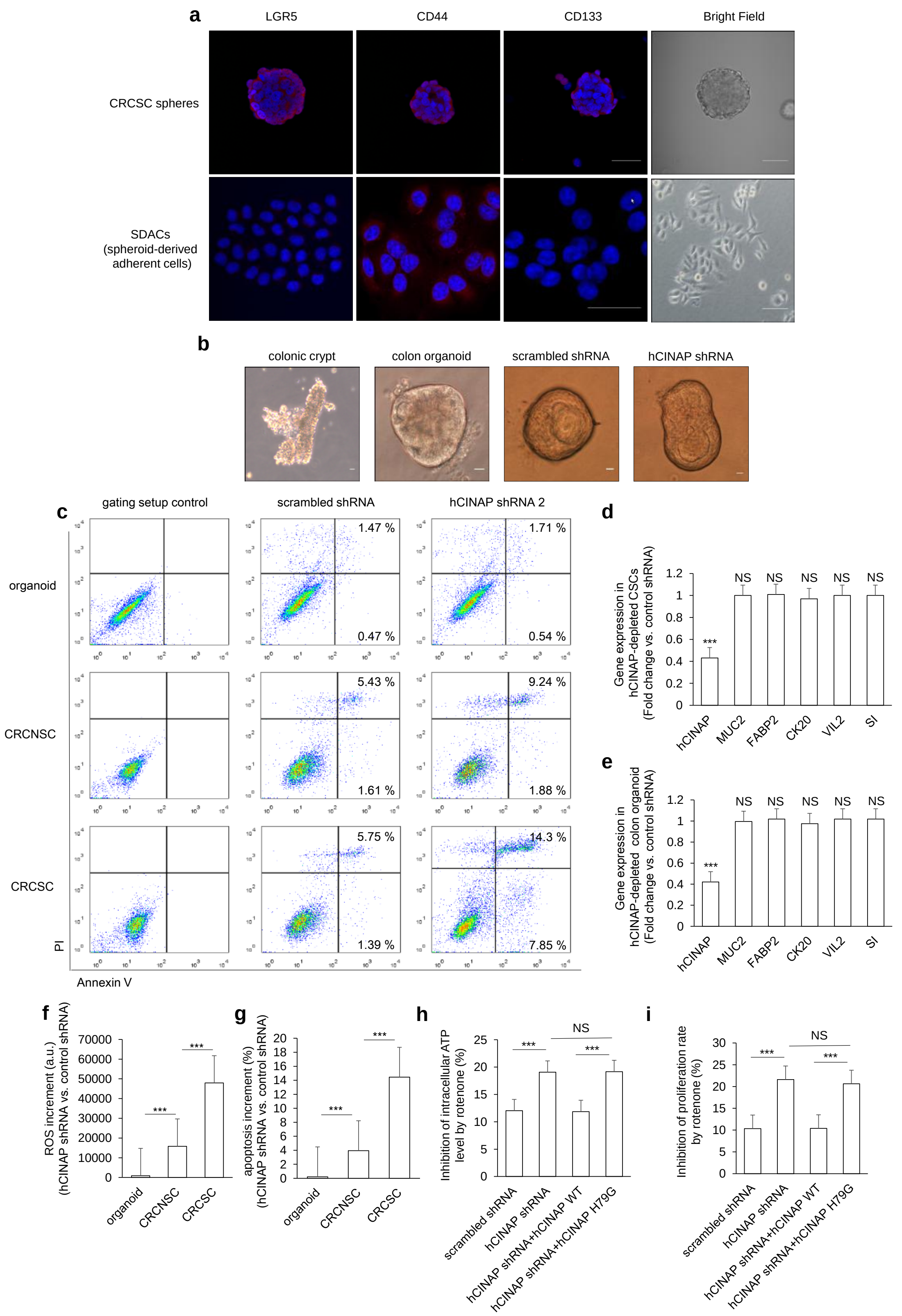

Supplementary Figure 5. Depletion of hCINAP affects apoptosis but not differentiation of CRCSCs. (**a**) Immunofluorescence analysis of patient-derived CRCSC (colorectal cancer stem cells) spheres and SDACs (sphere-derived adherent cells, differentiated from CRCSCs). CRCSC markers

LGR5, CD44 and CD133 were labeled in red and the nuclei were stained with DAPI (blue). The scale bar represents 200 μm. (**b**) Colon crypts isolated from normal colon tissue and cultured in matrigel, transfected with hCINAP or control shRNA. Scale bar, 200 μm. (**c**) FACS analysis of apoptosis rate of organoid, CRCSCs and CRCNSCs (colorectal cancer non-stem cells), transfected with hCINAP or control shRNA. (**d**) and (**e**) The expressionof colon differentiation markers in hCINAP-depleted or RNAi control organoid and CRCSCs revealed by RT-qPCR analyses. The experiments were performed three times. (**f**) and (**g**) The increment of ROS level and apoptosis rate caused by hCINAP depletion in organoid, CRCSCs and CRCNSCs. The increments were determined by subtracting the ROS levels (or apoptosis rates) in cells treated with control shRNA from the values in cells treated with hCINAP shRNA. The unit of ROS (a.u.) is short for arbitrary unit. (**h**) and (**i**) The inhibition of intracellular ATP level and proliferation rate by rotenone (50 nM) in SW480 cells transfected with indicated plasmids. The increments were determined by subtracting the ATP levels (or proliferation rates) in cells treated with DMSO from the values in cells treated with rotenone. Data in (**d-i**) are presented as mean ± SEM (N=3). NS, not significant, ***p<0.001.

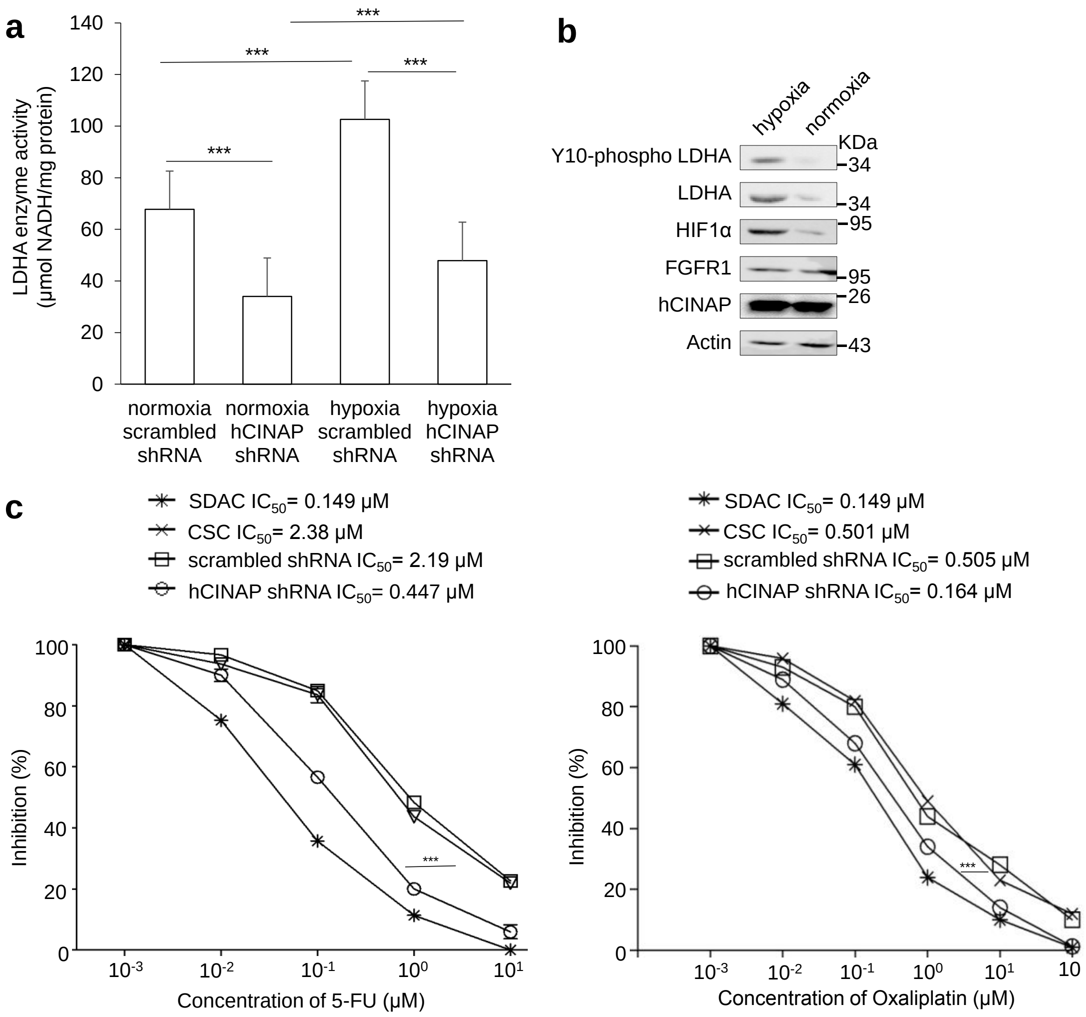

Supplementary Figure 6. hCINAP contributes to CRCSC stemness underhypoxia. (**a**) Measurement of LDHA activity in CRCSCs stably transfected with hCINAP or control shRNA, under hypoxia or normoxia. (**b**) Western blot analyses of LDHA Y10 phosphorylation, LDHA and HIF1α levels under hypoxia and normoxia. (**c**) Measurement of the viable cell inhibition rate resulted from 5-FU and Oxaliplatin in CRCSCs and the results were analyzed by Bonferroni correction,***p<0.001.SDAC indicates spheroid-derived adherent cells, and CSC indicates cancer stem cell.

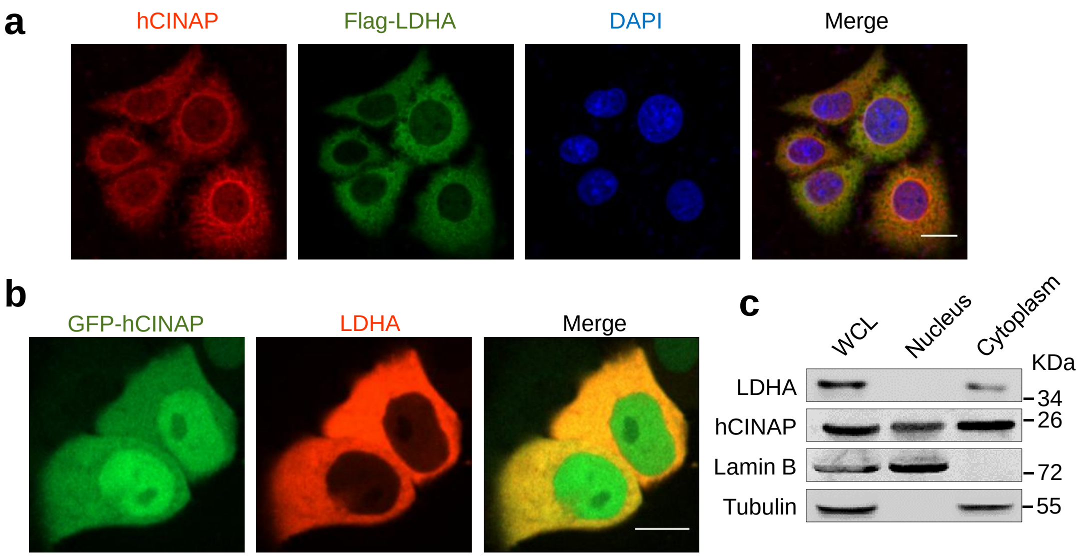

Supplementary Figure 7. hCINAP is localized in the cytoplasm and the nuclear. (a) and **(b)** Subcellular localization of endogenous hCINAP, Flag-LDHA, GFP-hCINAP and endogenous LDHA protein in SW480 cells was visualized by immunofluorescence microscopy using the hCINAP and LDHA antibody (red), Flag antibody (green) and DAPI (blue). Scale bar, 10 μm. (**c**) Cell fraction analysis of hCINAP and LDHA in SW480 cells. Fractions of the cytoplasm and the nucleus, and WCL (whole cell lysate) were detected by Western blotting using the hCINAP and LDHA antibodies. Tubulin and Lamin B were used as markers for the cytoplasm and the nuclear, respectively.

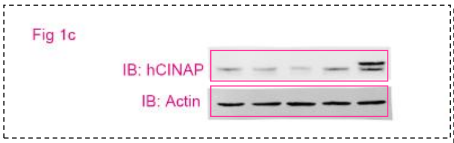
Fig 1c
IB: hCINAP
IB: Actin

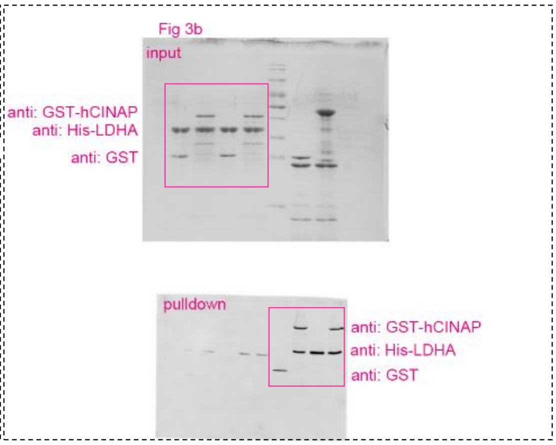
Fig 3b
input
anti: GST-hCINAP
anti: His-LDHA
anti: GST
pulldown
anti: GST-hCINAP
anti: His-LDHA
anti: GST

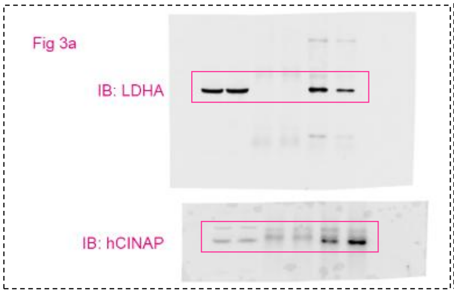
Fig 3a
IB: LDHA
IB: hCINAP

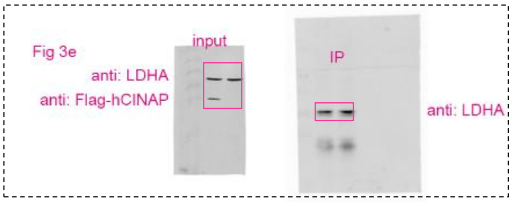
Fig 3e
input
anti: LDHA
anti: Flag-hCINAP
IP
anti: LDHA

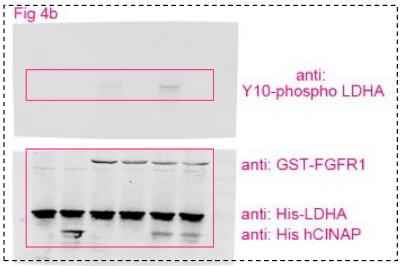
Fig 4b
anti: Y10-phospho LDHA
anti: GST-FGFR1
anti: His-LDHA
anti: His hCINAP

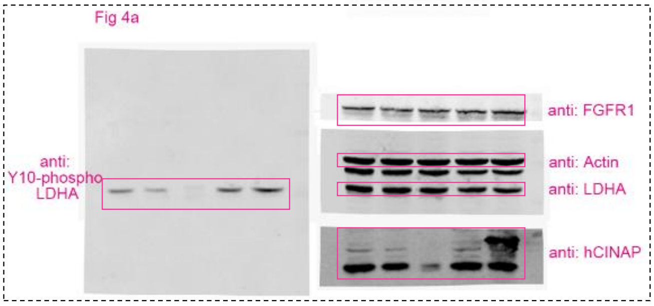
Fig 4a
anti: Y10-phospho LDHA
anti: FGFR1
anti: Actin
anti: LDHA
anti: hCINAP

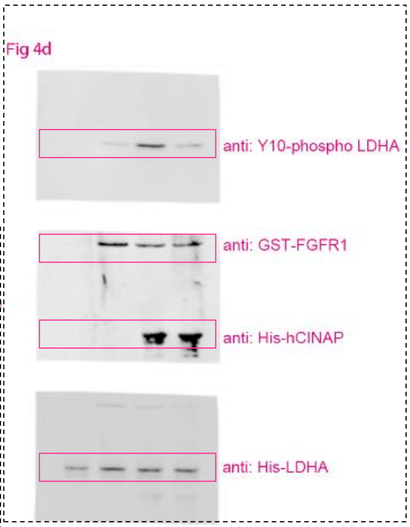
Fig 4d
anti: Y10-phospho LDHA
anti: GST-FGFR1
anti: His-hCINAP
anti: His-LDHA

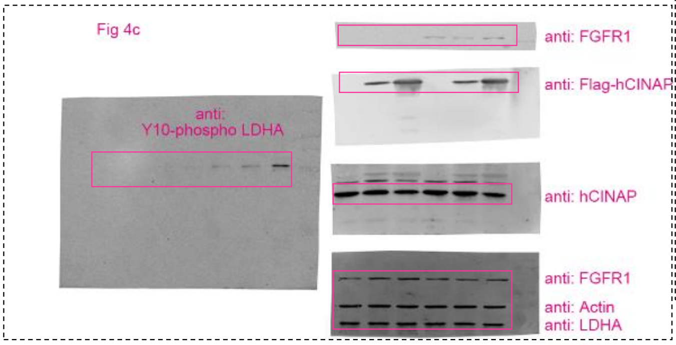
Fig 4c
anti: Y10-phospho LDHA
anti: FGFR1
anti: Flag-hCINAP
anti: hCINAP
anti: FGFR1
anti: Actin
anti: LDHA

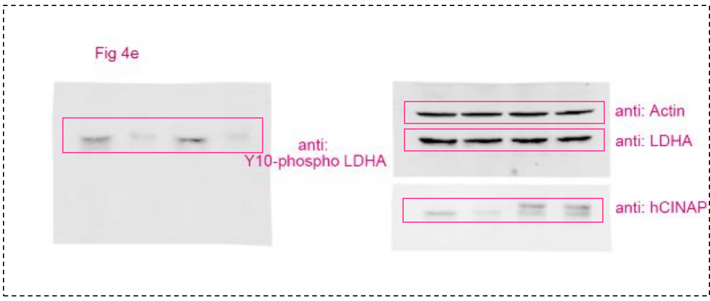
Fig 4e
anti: Y10-phospho LDHA
anti: Actin
anti: LDHA
anti: hCINAP

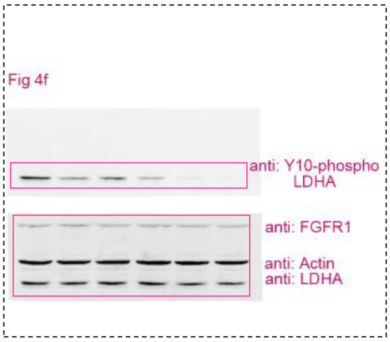
Fig 4f
anti: Y10-phospho LDHA
anti: FGFR1
anti: Actin
anti: LDHA

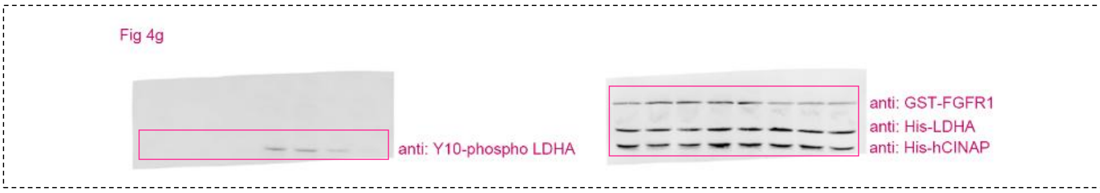
Fig 4g
anti: Y10-phospho LDHA
anti: GST-FGFR1
anti: His-LDHA
anti: His-hCINAP

Fig 5b

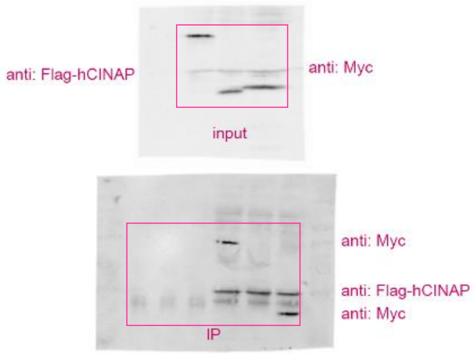

Fig 5c

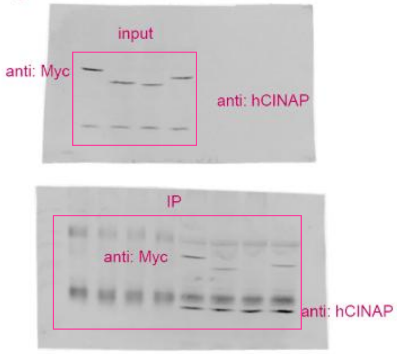

Fig 5d

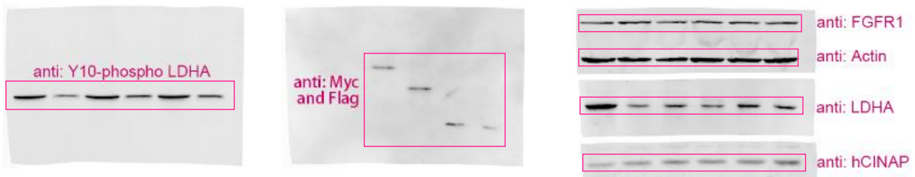

Fig 5g

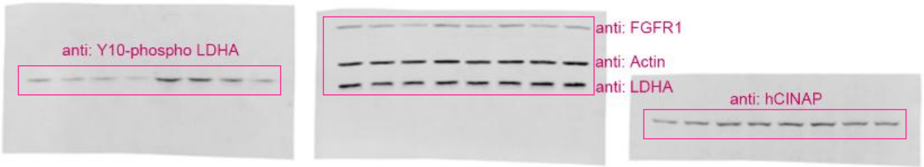

Fig 5h

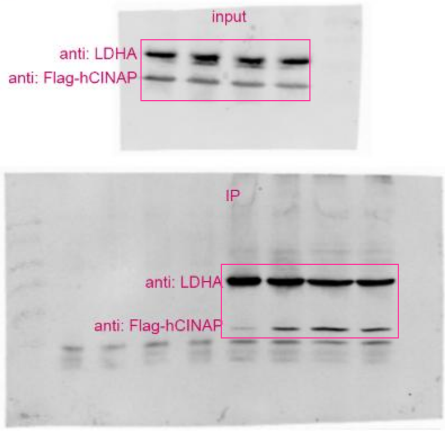

Fig 5i

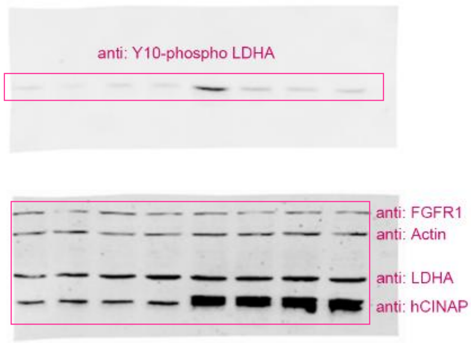

Fig 6a & Fig 7d

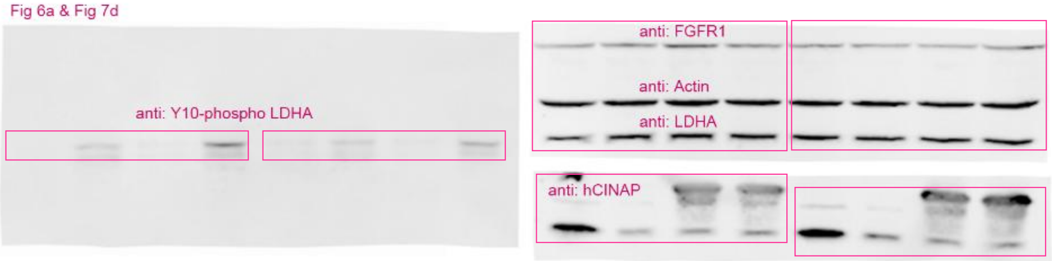

Fig 6h

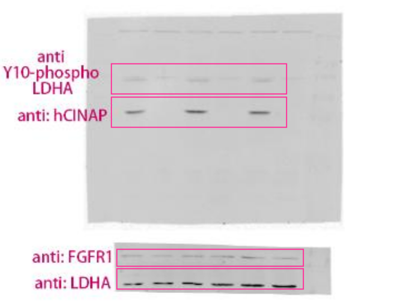

Fig 7c

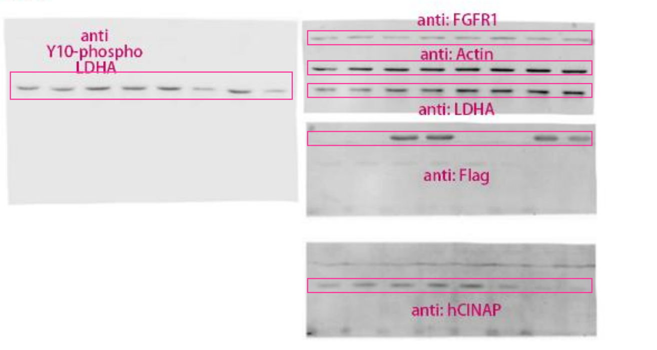

Supplementary Fig 1a

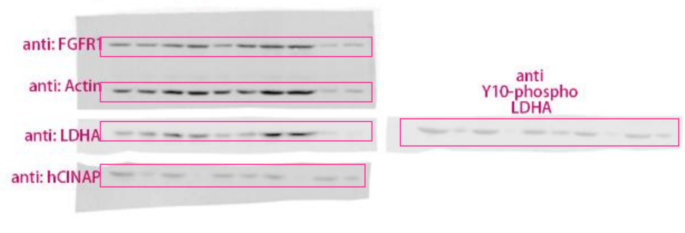

Supplementary Fig 2a

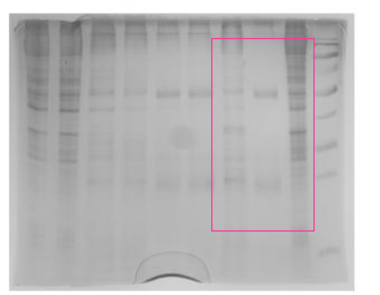

Supplementary Fig 3a

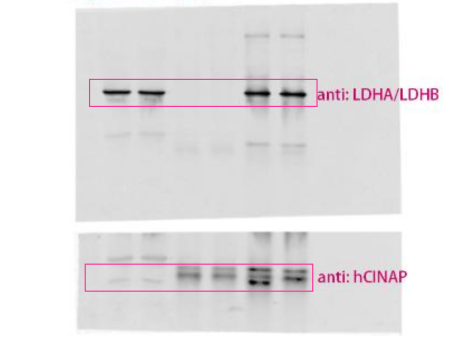

Supplementary Fig 3b

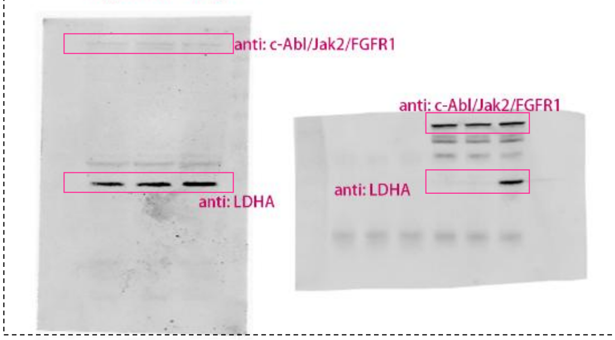

Supplementary Fig 3c

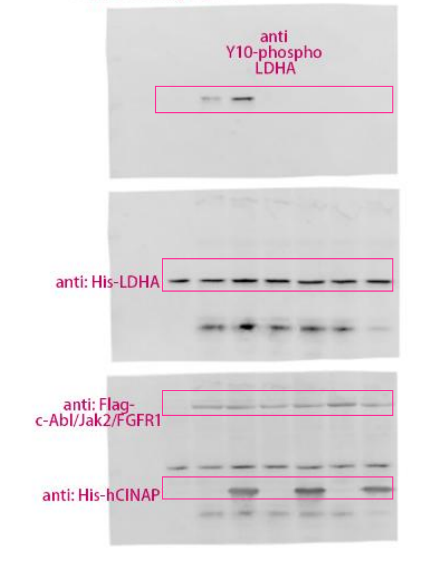

Supplementary Fig 3d

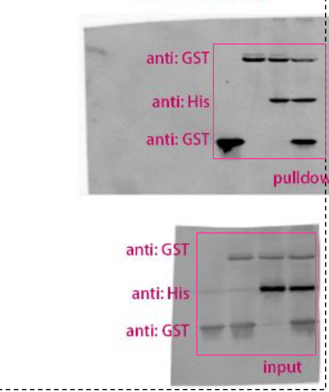

Supplementary Fig 3e

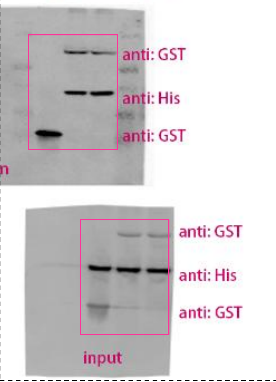

Supplementary Fig 3f

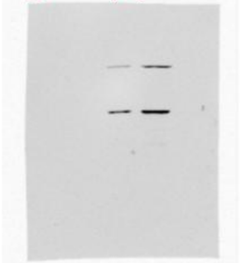

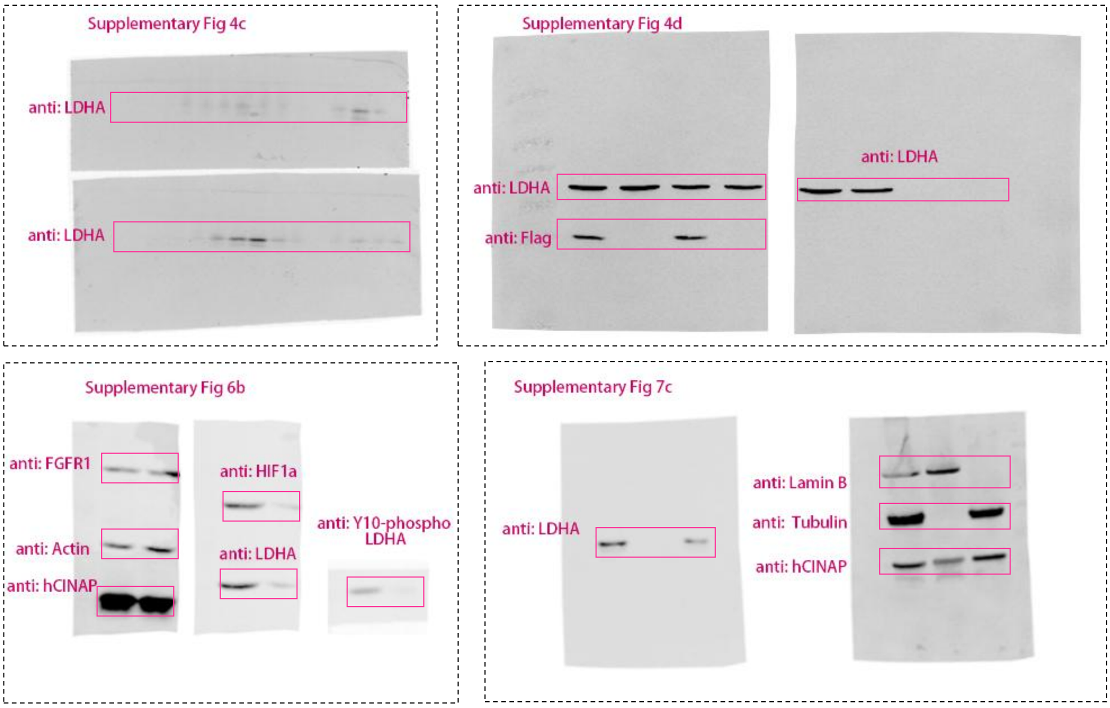

Supplementary Figure 8. Uncropped scans of the most important blots. Red boxes are the cropped bands in the indicated figures.

Supplementary Table 1. List of primers used for real time q-PCR

gene	Forward primer	Reverse primer
CD133	CCCCGCAGGAGTGAATCTTT	GAAGGACTCGTTGCTGGTGA
LGR5	GTTTCCCGCAAGACGTAACT	CAGCGTCTTCACCTCCTACC
ALDH1A1	ATCAAAGAAGCTGCCGGGAA	GCATTGTCCAAGTCGGCATC
CD26	AGGCACAGATGATGCTACAG	GCCTCCATTGCTTCACGTAG
Bmi1	CTGTATGCCTAAAAGCGGGT	ATCTGCAAAGGTCGAACCAG
Dclk1	TGGACTACTACCAGCAACCA	TAAAAGGGCGAGTTAGGGGA
E-cadherin	CTTTGACGCCGAGAGCTACA	TTTGAATCGGGTGTCGAGGG
α-catenin	GGCAGCCAAAAGACAACAGG	GGCCTTATAGGCTGCGACAT
γ-catenin	CAACCAGGAGAGCAAGCTGA	CCTCCACAATGGCAGGCTTA
Fibronectin	CTGGCCAGTCCTACAACCAG	CGGGAATCTTCTCTGTCAGCC
Vimentin	GGACCAGCTAACCAACGACA	AAGGTCAAGACGTGCCAGAG
TWIST1	TCTACCAGGTCCTCCAGAGC	CTCCATCCTCCAGACCGAGA
SNAIL1	CGGAAGCCTAACTACAGCGA	GCCAGGACAGAGTCCCAGAT
MMP2	GTCGCCCATCATCAAGTTCC	CGCATGGTCTCGATGGTATT
MMP9	CCTGGGCAGATTCCAAACCT	GTACACGCGAGTGAAGGTGA
MMP10	TTCCTGACGTTGGTCACTTC	TAGGCATGAGCCAAACTGTG
VIL2	TGGTAAAGACTATCGGCCT	GGCAGTAGATCTCATCGC
SI	CCCCATTCAAGAACCAGAT	GTAACACTGTCTGTCAACC
CK20	CACACGGTGAACTATGGG	CCGAGCATTTTGCAGTTG
FABP2	AAGGTAGACCGGAGTGAAA	GTAACACTGTCTGTCAACC
MUC2	GCCCATCTATGAGGAGGA	GTTGAAGTGCTTCTCCAC
GAPDH	AAGGTGAAGGTCGGAGTC	AATGAAGGGGTCATTGATG

Supplementary Table 2. Patient and tumor characteristics

Patient number	Age	Sex	Tumor site	State	Grade
1	89	M	Right half colon	2A	II-III
2	80	M	Transverse colon	2A	II-III
3	74	M	Descending colon	3	II
4	60	F	Transverse colon	2A	II-III
5	71	F	Ascending colon	1	II-III
6	73	F	Right half colon	3B	III
7	62	F	Ascending colon	3	II
8	62	F	Sigmoid	2A	II
9	65	M	Sigmoid	2A	II
10	48	M	Left half colon	2C	III
11	82	F	Sigmoid	1	I
12	24	F	Transverse colon	3B	II-III
13	73	M	Sigmoid	4A	I
14	78	M	Sigmoid	1	I-II
15	80	M	Ascending colon	2A	II
16	84	F	Ascending colon	3B	II
17	73	M	Sigmoid	2A	II-III
18	71	M	Ascending colon	2A	II
19	70	F	Sigmoid	3B	II-III
20	70	F	Right half colon	2A	II
21	62	F	Ascending colon	3C	III
22	62	M	Sigmoid	2A	II
23	58	F	Sigmoid	3B	II-III
24	75	M	Right half colon	3B	II
25	80	M	Right half colon	3B	III
26	79	F	Ascending colon	3B	II
27	90	M	Right half colon	2A	II-III
28	67	F	Sigmoid	3B	II-III
29	58	M	Sigmoid	2A	I-II
30	81	F	Sigmoid	2A	II
31	74	F	Ascending colon	1	II
32	66	M	Right half colon	2A	II-III
33	53	F	Descending colon	2A	II
34	67	M	Left half colon	3B	II-III
35	84	F	Sigmoid	1	II
36	67	M	Splenic flexure of colon	2A	II-III
37	65	F	Right half colon	3B	II
38	64	M	Sigmoid	2A	II-III
39	55	M	Sigmoid	2A	I-II
40	47	M	Ascending colon	1	I

41	63	F	Sigmoid	3B	II
42	64	F	Sigmoid	3B	I
43	63	M	Splenic flexure of colon	2A	II
44	58	F	Sigmoid	2A	II
45	75	F	Sigmoid	2A	II
46	57	M	Hepatic flexure of colon	3B	II
47	53	M	Splenic flexure of colon	2A	II-III
48	76	F	Sigmoid	2A	II
49	78	M	Rectum	2A	II
50	85	M	Right half colon	3	II-III
