# Supplementary material for: Adenylate kinase hCINAP determines self-renewal of colorectal cancer stem cells by facilitating LDHA phosphorylation
Source: Nat Commun. 2017 May 18;8:15308. doi: 10.1038/ncomms15308 (PMC5454382; doi:10.1038/ncomms15308)
Supplement: Peer Review File [file ncomms15308-s2.pdf]

Reviewers' comments:

Reviewer #1 (Remarks to the Author):

Major claims of the paper:
The authors reported findings describing that an atypical adenylate kinase hCINAP is overexpressed in colorectal cancer, while knockdown of hCINAP attenuates the invasive and self renewal potential of CRC stem cells, leading to reduced tumor growth. The authors found that hCINAP interacts with glycolytic enzyme LDHA and promotes LDHA Y10 phosphorylation, which requires adenylate kinase activity. Y10 phosphorylation has been reported to be commonly upregulated in human cancers to enhance LDHA enzyme activity and promotes glycolysis, thus the authors' findings suggest a mechanistic link between hCINAP upregulation and lactate production that provides a metabolic advantage for acidic microenvironmental that is benefitial to CRC stem cell invasion. The authors strengthened their model by providing physiological impact showing that hCINAP expression correlates with Y10-phosphorylation levels of LDHA in primary tumor tissue samples from CRC patients.

Are they novel and will they be of interest to others in the field?
The findings are interesting and novel. hCINAP is a nuclear ATPase/adenylate kinase and has been reported to be involved in regulation of ribosomal RNA and protein. The current manuscript is the first report showing its potential role in regulation of cancer metabolism and provides some interesting mechanistic insights. Therefore, this reviewer thinks that the findings in the paper will be of interest to a wide audience.

Is the work convincing, and if not, what further evidence would be required to strengthen the conclusions?
Overall, the studies are relatively well designed, conducted and controlled. The quality of data is excellent. This reviewer has several major concerns for the authors to address before the paper can be further considered by the journal:

(1) hCINAP has been reported as a nuclear protein so how is hCINAP recruited to cytosol to interact with LDHA? Or in which subcellular compartments (cytosol or nucleus) does such interaction occur and function to promote the Warburg effect in CRCSC?
(2) Figure 1b should include more controls including enzyme dead mutants of hCINAP. Figure 1 needs Western blot control to show expression of FLAG-hCINAP proteins in cells.
(3) Figure 3 needs more metabolic assays to determine whether knockdown of hCINAP would inhibit glycolysis by attenuating LDHA and promotes OXPHOS. Or the cells are still glycolytic but now rely on mitochondrial complex I for NAD+ to sustain glycolysis, if so the cells with hCINAP knockdown should be more sensitive to treatment with complex I inhibitor rotenone in terms of inhibition of cell proliferation rate and ATP levels. Similar concerns apply to Figure 6 as well.
(4) In Figure 4, it is not clear whether the effect of adenylate kinase in facilitating LDHA Y10 phosphorylation is specific to FGFR1, or other upstream oncogenic tyrosine kinases of LDHA including JAK2 and BAL as reported would also benefit from adenylate kinase-dependent conversion of 2xADP to ATP and AMP? Also it is not clear whether these three proteins could form a protein complex and adenylate kinase binding would facilitate substrates (LDHA and ATP) recruitment to upstream tyrosine kinase? Does the enzyme dead mutant of hCINAP still bind to LDHA?
(5) It is also not clear whether the binding between adenylate kinase and LDHA is required for AK effect on LDHA. It will be informative if the authors could identify the binding region of LDHA and generate LDHA mutants that are deficient of nCINAP binding. Expression of such binding-deficient LDHA in cells with stable knockdown of LDHA should not be able to rescue the decreased cell proliferation and glycolysis compared to cells with rescue expression of control LDHA wild type.
(6) In Figure 7 the authors also need to provide more control results, they need to show, for example, at least Y10 phosphorylation levels of LDHA in tumors derived from cells with hCINAP knockdown, and whether rescue expression of WT or H79G would affect Y10 phosphorylation of

LDHA in these cells

On a more subjective note, do you feel that the paper will influence thinking in the field?
Yes this is a quite novel finding that is surprising. The proposed mechanism describes an interesting role of adenylate kinase in facilitating protein phosphorylation, which provides new insights to advance our understanding of protein phosphorylation.

Comment on the appropriateness of any statistical tests, and the accuracy of the description of any error bars and probability values
No major concerns

Reviewer #2 (Remarks to the Author):

This is a very interesting paper that is topical and of considerable interest. The studies have been conducted well although I have one significant concern regarding figure 1a. This figure presents the results of western blot analysis and concludes that hCINAP expression is elevated in CRC compared to adjacent normal tissue. This is too strong a conclusion based on one set of data and it requires (i) immune-histochemical studies to determine heterogeneity within and between tumours (ii) details of the tumours used in the study (i.e.pathological grade/stage and what treatment these patients may have had etc) (iii) stem cells are typically associated with the hypoxic niche and this is potentially important in the context of this study (energy production and EMT). This will require further work to strengthen this aspect of the publication.

The remaining issues are comparatively minor and these are listed below

(i) Page 5, line 103: Are the cells used primary cultures or have they been passaged several times in which case they are effectively established lines?
(ii) Page 7, line 148: insert a gap between cellular and components
(iii) Page 8, line 157: I understand why the focus has been placed on LDH-A but it could be argued that LDH-B is also an important consideration, particularly in the context of the reverse Warburg effect as proposed by Lisanti (there is some mention of CAFs in the discussion but no link/discussion of the potential implications here for metabolic interactions between tumour/stroma). Is there any information about the effects of hCINAP on LDH-B?
(iv)Page 9, line192: Its AP5A in the text but 5 is subscript in the figures
(v) Page 13, line 261: The word dramatically is too bold here - its increased for sure but I would not call it dramatic.
(vi) Figures: As a general comment on all the figures, the annotations/axis labels appear blurred even on the on screen pdf version of the manuscript. This makes it hard to read. Furthermore the figure legends don't always tell the reader what the experimental conditions are (for example, what is SDAC and TIC in figure 2f? You can work it out but my feeling was that the ability to 'read' the p[aper would be enhanced if these conditions were defined in the legend.
(vii) Figure 5b and c: The text debating conditions in each lane are hard to read as there is no or a very small gap in the text.

Reviewer #3 (Remarks to the Author):

In this manuscript, the authors present evidence to suggest a role for CINAP (AK6) in regulating colorectal cancer stemness. Patient data revealed that CINAP expression is increased in CRC tissues compared to adjacent normal mucosae. The authors subsequently functionally test the role of CINAP in HCT116 by a series of knock down and overexpression experiments. They conclude that CINAP regulates key features of cancer stemness, such as clonogenic potential, invasive

capacity and therapy resistance. Mechanistically, CINAP seems to regulate LDHA activity, at least in part by facilitating LDHA phosphorylation at key residues by FGFR1. This presumably results in an increase in glycolysis and cancer stem cell phenotype. Although the manuscript contains some interesting parts, the role of FGFR1 in regulating LDHA has previously been described (REF: 21969607), hence the novelty around this section is only incremental. In addition, the choice of the cancer cell line HCT116 is not suitable for most experiments, as it has been shown not to follow the CSC model (PMID: 9294210). They should use another CRC line to evaluate the impact of CINAP on CSC function. Subsequently, the effect on CSCs should be delineated further. Are CSCs dying or differentiating into other lineages upon CINAP inhibition? If so, why are these cells more dependent on CINAP/LDHA/Glycolysis? These data would anchor the manuscript. Finally, they are also encouraged to make use of inducible knock down constructs for their in-vivo experiments to rule out engraftment artifacts. See below more detailed comments:

1) The CRC patient set should be described in more details. Is there any correlation of CINAP with disease stage or any other clinical parameter? Recent identification of molecular subsets of CRC has revealed a subtype that display poor prognosis and increased invasive features. Given the wealth of publically available data around this topic, the authors should investigate CINAP expression in various datasets/subtypes. Figure 1b/c should be corrected for proliferation effects.

2) Figure 2. An isotype control is warranted to setup gating. The increase of CD133/Lgr5 double positive is of note but seems at odd when clonogencity is evaluated by limiting dilution. A ~8 fold increase in the fraction of CSCs only results in a modest ~1.4 fold increase in clonogenicity, this data is unclear. Minor: the mesenchymal/epithelial panel of genes in 2d is inverted.

3) The authors should rule out that CINAP inhibition mediates a more general cancer cell growth inhibition that CSC specific. FACS sort of CD133 pos/neg cells could be done to correlate expression of CINAP, LDHA activity etc. Indeed, stem cell genes and EMT are affected but this doesn’t necessarily imply that a stem cell pool is altered.

4) Because CINAP has been shown recently shown to regulate cancer cell growth by regulating rRNA processing, it is important to specifically demonstrate the dependency of LDHA for mediating CINAP effect on cancer stemness. For instance, can CINAP overexpression rescue LDHA KD? Conversely, normal mucosae still display CINAP expression (figure 1a) and a positive correlation between CINAP and phosphor Y10 LDHA. Are the authors anticipating gut toxicity from CINAP inhibition in healthy tissue as well? Could an organoid culture from human colon be established to test this?

5) As mentioned above, in figure 7 the lack of tumor take could be a reflection of poor engraftment or low viability of engrafted cells, altogether making the clinical relevance of the study unclear. Inducible KD would significantly lift the impact of the current manuscript.

Reviewers' comments:

Reviewer #1 (Remarks to the Author):

Major claims of the paper:

The authors reported findings describing that an atypical adenylate kinase hCINAP is overexpressed in colorectal cancer, while knockdown of hCINAP attenuates the invasive and self renewal potential of CRC stem cells, leading to reduced tumor growth. The authors found that hCINAP interacts with glycolytic enzyme LDHA and promotes LDHA Y10 phosphorylation, which requires adenylate kinase activity. Y10 phosphorylation has been reported to be commonly upregulated in human cancers to enhance LDHA enzyme activity and promotes glycolysis, thus the authors' findings suggest a mechanistic link between hCINAP upregulation and lactate production that provides a metabolic advantage for acidic microenvironmental that is benefitial to CRC stem cell invasion. The authors strengthened their model by providing physiological impact showing that hCINAP expression correlates with Y10-phosphorylation levels of LDHA in primary tumor tissue samples from CRC patients.

Are they novel and will they be of interest to others in the field?

The findings are interesting and novel. hCINAP is a nuclear ATPase/adenylate kinase and has been reported to be involved in regulation of ribosomal RNA and protein. The current manuscript is the first report showing its potential role in regulation of cancer metabolism and provides some interesting mechanistic insights. Therefore, this reviewer thinks that the findings in the paper will be of interest to a wide audience.

Is the work convincing, and if not, what further evidence would be required to strengthen the conclusions?

Overall, the studies are relatively well designed, conducted and controlled. The quality of data is excellent. This reviewer has several major concerns for the authors to address before the paper can be further considered by the journal:

(1) hCINAP has been reported as a nuclear protein so how is hCINAP recruited to cytosol to interact with LDHA? Or in which subcellular compartments (cytosol or nucleus) does such interaction occur and function to promote the Warburg effect in CRCSC?

Response:

To address this comment, we performed Immunofluorescence and Subcellular fractionation assays to detect the subcellular localization of hCINAP. Our immunofluorescence results showed that endogenous hCINAP protein was localized mainly in the cytosol (Supplementary Figure 7a), while exogenously expressed GFP-tagged hCINAP was localized more in the nucleus (Supplementary Figure 7b). The results of subcellular fractionation assays indicated that hCINAP was localized in both cytosol and nucleus (Supplementary Figure 7c). It is the final step of glycolysis that pyruvate is converted into lactate by LDHA in cytosol, and we found that LDHA was co-localized and interacted

with hCINAP in cytosol (Supplementary Figure 7c and Figure 3a), indicating that hCINAP interacts with LDHA to promote glycolysis in the cytosol of CRCSC. Taken together, cytosol-localized hCINAP interacts with LDHA and upregulates LDHA activity, and promotes the Warburg effect in CRCSC. We added the data as supplementary data (Supplementary Figure 7 and Figure 3a) and presented it on page 8.

(2) a. Figure 1b should include more controls including enzyme dead mutants of hCINAP.

Response:

CRCSCs have been suggested to have an important role during the invasive and metastatic processes (*Nat Rev Cancer.* 2005, **5**, 899-904). Following the suggestion from the reviewer, we performed migration assays to verify whether adenylate kinase activity of hCINAP is requisite for CRC invasion by using hCINAP or control shRNA and rescue expression of hCINAP WT or H79G enzyme dead mutant in CRCSCs (Figure 7e). And migration potential of cells was normalized to cell proliferation rate to warrant that the migration potential was just influenced excessively by cell growth. Consistent to the result of Figure 1b, migration assays showed that wild type hCINAP, instead of hCINAP H79G mutant, promoted CRC migration. We provided this result in Figure 7e in the revision.

b. Figure 1 needs Western blot control to show expression of FLAG-hCINAP proteins in cells.

Response:

As suggested by the reviewer, western blot analysis of the expression of FLAG-hCINAP was shown in Figure 1c in the updated version.

(3) Figure 3 needs more metabolic assays to determine whether knockdown of hCINAP would inhibit glycolysis by attenuating LDHA and promotes OXPHOS. Or the cells are still glycolytic but now rely on mitochondrial complex I for NAD^+ to sustain glycolysis, if so the cells with hCINAP knockdown should be more sensitive to treatment with complex I inhibitor rotenone in terms of inhibition of cell proliferation rate and ATP levels. Similar concerns apply to Figure 6 as well.

Response:

It was documented that H1299 lung cancer cells were highly glycolytic, while H1299 cells with depleted LDHA and LDHA Y10F mutant rescue expression were still glycolytic, but was more dependent on mitochondrial NADH:ubiquinone oxidoreductase (Complex I) for NAD^+ generation to maintain glycolysis (*Nat Rev Cancer.* 2005, **5**, 899-904). To test whether hCINAP enzymatic activity had a similar effect on LDHA activity in regulating cell metabolism, we measured Extracellular Acidification Rate (ECAR) and O_2 Consumption Rate (OCR) in CRCSCs. The results of ECAR and OCR measurement showed that both rescue expression of hCINAP stably knockdown and hCINAP H79G mutant inhibited glycolysis (Figure 6d,e) and enhanced mitochondrial respiration (Figure 6f,g) in comparsion to those of the control CRCSCs. These results indicate that hCINAP promotes glycolysis and suppresses OXPHOS by facilitating LDHA Y10 phosphorylation in CRCSCs. Meanwhile, the proliferation rate of cells with knocked down hCINAP or hCINAP H79G mutant rescue were more sensitive to rotenone treatment (Supplementary Figure 5h,i), suggesting that

hCINAP-depleted cells rely more on mitochondrial complex I for NAD^+ production to survive. We added the new data in the revision in Figure 6d,e,f,g,k and Supplementary Figure 5h,i.

(4) In Figure 4, it is not clear whether the effect of adenylate kinase in facilitating LDHA Y10 phosphorylation is specific to FGFR1, or other upstream oncogenic tyrosine kinases of LDHA including JAK2 and BAL as reported would also benefit from adenylate kinase-dependent conversion of 2xADP to ATP and AMP? Also it is not clear whether these three proteins could form a protein complex and adenylate kinase binding would facilitate substrates (LDHA and ATP) recruitment to upstream tyrosine kinase? Does the enzyme dead mutant of hCINAP still bind to LDHA?

Response:

It was reported that multiple oncogenic tyrosine kinases phosphorylated LDHA at Y10 in various cancers, such as FGFR1 in lung cancer, BCR-Abl in chronic myelogenous leukemia and JAK2 in human erythroleukemia. To test whether FGFR1 is the specific kinase responsible for LDHA Y10 phosphorylation in CRC, we first performed Co-IP assay to analyze the interaction of LDHA with these three tyrosine kinases. The results showed that LDHA only interacted with FGFR1 in SW480 cells (Supplementary Figure 3b). In addition, *in vitro* kinase assays showed that FGFR1, but not c-Abl (no BCR-Abl translocation in SW480 cells) or JAK2, directly phosphorylated LDHA at Y10 facilitated by hCINAP (Supplementary Figure 3c). Therefore, the effect of adenylate kinase in facilitating LDHA Y10 phosphorylation is specific to FGFR1, indicating that the tyrosine kinase responsible for LDHA Y10 phosphorylation is cancer cell type specific.

To identify whether FGFR1, LDHA and hCINAP form a protein complex, we conducted the *in vitro* GST Pull-down assay and found that FGFR1 had no direct interaction with hCINAP, while LDHA interacted with both FGFR1 and hCINAP, suggesting that these three proteins form a ternary complex, the interaction between hCINAP and FGFR1 was intermediated by LDHA, and hCINAP did not affect the interaction between FGFR1 and LDHA (Supplementary Figure 3d). Moreover, *in vitro* FGFR1 kinase assay by using γ-^{32}P labeled ATP revealed that hCINAP enhanced the ATP-FGFR1 radiolabelling signal, indicating that hCINAP enhanced ATP recuitment to FGFR1 (Supplementary Figure 3f).

Following the suggestion from the reviewer, we also performed GST Pull-down assay to test whether the enzyme dead H79G mutant affected the hCINAP-LDHA interaction. The result showed that both hCINAP wild-type and H79G mutant associated with LDHA (Supplementary Figure 3e), implying that hCINAP enzyme dead mutant still bind to LDHA.

We added the results above in Supplementary Figure 3 and discussed it in the revision.

(5) It is also not clear whether the binding between adenylate kinase and LDHA is required for AK effect on LDHA. It will be informative if the authors could identify the binding region of LDHA and generate LDHA mutants that are deficient of hCINAP binding. Expression of such binding-deficient LDHA in cells with stable knockdown of LDHA should not be able to rescue the decreased cell proliferation and glycolysis compared to cells with rescue expression of control LDHA wild type.

Response:

To clarify whether the binding between adenylate kinase and LDHA is required for AK effect on LDHA, we constructed various LDHA truncations containing the N-terminal catalytic domain (1-160 amino acids) and C-terminal non-catalytic domain (161-332 amino acids) , respectively (Figure 5a), and performed Co-IP assays to analyze their interaction with hCINAP. Co-IP results showed that LDHA C-terminal domain interacted with hCINAP (Figure 5b). Furthermore, we divided LDHA C-terminal domain into three parts based on its secondary structure and constructed the corresponding LDHA C-terminal deletion mutants (LDHA161-218, 219-278 and 279-332 deletion mutants) (Figure 5a) for Co-IP analyses. The data exhibited that 219-278 deletion abolished the LDHA-hCINAP interaction (Figure 5c), implying that amino acids 219-278 were critical for the binding of LDHA to hCINAP.

As hCINAP mainly affected invasion, self-renewal and tumorigenesis in a glycolysis-dependent manner by facilitating LDHA phosphorylation, next, we verified whether the abrogation of the LDHA-hCINAP interaction affected the phenotypes of CRCSCs, by introducing Δ219-278 deletion mutant into CRCSCs (Figure 5d,e,f). The result of LDHA phosphorylation assay showed that knockdown of LDHA reduced LDHA phosphorylation significantly; the level of LDHA phosphorylation could only be rescued by LDHA or wild type hCINAP but not LDHA Δ219-278 or hCINAP H79G mutant (Figure 5d). Similarly, Immunoflueréscence results showed that LDHA depletion by shRNA caused inhibition of EMT in CRCSC spheres, while rescue expression of LDHA wild-type, but not Δ219-278 deletion mutant, restored the EMT phenotype of CRCSCs. We also found that rescue expression of hCINAP wild-type, instead of its H79G mutant, recovered EMT in CRCSCs (Figure 5e). We obtained the consistent data in tumorsphere formation assay (Figure 5f). Taken together, we concluded that the LDHA-hCINAP interaction is requisite for the function of hCINAP in the Warburg effect and stemness maintenance in CRCSCs.

We added the results above in Figure 5a-f and discuss them in this revision.

(6) In Figure 7 the authors also need to provide more control results, they need to show, for example, at least Y10 phosphorylation levels of LDHA in tumors derived from cells with hCINAP knockdown, and whether rescue expression of WT or H79G would affect Y10 phosphorylation of LDHA in these cells.

Response:

As suggested by the reviewer, the level of Y10 LDHA phosphoryaltion, hCINAP and Actin in CRCSCs with hCINAP knockdown and rescue expression of hCINAP wild-type and H79G mutant was shown in the updated manuscript (Figure 7d). Indeed, hCINAP knockdown inhibited LDHAY10 phosphoryaltion compared to that in control shRNA-treated CRCSCs. And hCINAP wild-type, instead of hCINAP H79G mutant rescued LDHA Y10 phosphotylation in CRCSCs.

Reviewer #2 (Remarks to the Author):

This is a very interesting paper that is topical and of considerable interest. The studies have been conducted well although I have one significant concern regarding figure 1a. This figure presents the results of western blot analysis and concludes that hCINAP expression is elevated in CRC compared

to adjacent normal tissue. This is too strong a conclusion based on one set of data and it requires (i) immune-histochemical studies to determine heterogeneity within and between tumours (ii) details of the tumours used in the study (i.e.pathological grade/stage and what treatment these patients may have had etc) (iii) stem cells are typically associated with the hypoxic niche and this is potentially important in the context of this study (energy production and EMT). This will require further work to strengthen this aspect of the publication.

Response:

To address the comments from the reviewer, we performed the following experiments.

(i) We performed immunohistochemical staining to confirm the high expression of hCINAP in CRC. The results showed that hCINAP expression is elevated in CRC compared to adjacent normal tissues (Supplementary Figure 1c). And hCINAP expression levels were distinct within and between CRCs, indicating heterogeneity in CRC.

(ii) The detailed information about tumor tissues used in this research was shown in Supplementary Table 2 including age, sex, tumor site, pathological state and grade of the patients.

(iii) CSCs are metastatic cancer cells that reside in the hypoxic niche, protecting them from the immune system and facilitating their invasive potential. Hypoxia induces CSCs to express hypoxia-inducible factors, including HIF1α. HIF1α promotes EMT in CSCs through reactivation of oxygen species (ROS)-activited TFG-β and TNF-α signaling pathways (*Nat Rev Cancer.* 2005, **5**, 899-904; *Mol Cell Biol. 2011*, **31**, 4938-4950), which is a key transcription factor to promote glycolysis by up-regulating the expression of glycolysis enzymes, such as glucose transporters, hexokinase 2 and LDHA, and inhibits OXPHOS via enhancing PDK1 expression (*Mol Cell Biol. 2011*, **31**, 4938-4950). To investigate whether hypoxia affected CRCSCs potential facilitated by hCINAP, we performed limiting dilution assay and found that hCINAP duplicated the promotion effect on tumor-initiating potential of CRCSC by hypoxic niche (Figure 3h). Moreover, hCINAP had an addititive effect on LDHA activity enhanced by hypoxia, compared to normoxia (Supplementary Figure 6a). We also found that hypoxia promoted LDHA but not hCINAP expression (Supplementary Figure 6b). Taken together, hCINAP enhanced LDHA activity and hypoxia promoted LDHA expression to maintain stemness of CRCSCs.

These data were added in the revision Figure 3h, Supplementary Table 2, Supplementary Figure 1c, Figure 6a,b.

The remaining issues are comparatively minor and these are listed below

(i) Page 5, line 103: Are the cells used primary cultures or have they been passaged several times in which case they are effectively established lines?

Response:

Cells used here were primary cultures derived from CRC patients.

(ii) Page 7, line 148: insert a gap between cellular and components

Response:

We insert a gap as recommended.

(iii) Page 8, line 157: I understand why the focus has been placed on LDH-A but it could be argued that LDH-B is also an important consideration, particularly in the context of the reverse Warburg effect as proposed by Lisanti (there is some mention of CAFs in the discussion but no link/discussion of the potential implications here for metabolic interactions between tumour/stroma). Is there any information about the effects of hCINAP on LDH-B?

Response:

The reverse Warburg effect is referred to the metabolic-coupling between cancer cells and stromal components (mesenchymal stem cells or cancer associated fibroblasts) and has been demonstrated in multiple cancers (*Cell Cycle. 2009,* **8**, 3984-4001). Stromal components secrete lactate (or pyruvate) generated from aerobic glycolysis to feed cancer cells. In return, cancer cells supply ATP from oxidative phosphorylation for stroma. Contrary to LDHA, LDHB enhances the conversion from lactate to pyruvate to fuel oxidative phosphorylation (*Oncotarget. 2014,* **5**, 7982-7983). Although LDHB shares 75% identity of protein sequence with LDHA, we did not detect the interaction between LDHB and hCINAP (Supplementary Figure 3a), indicating that hCINAP had no impact on LDHB in CRC. Nevertheless, the Warburg effect facilitated by hCINAP in cancer cells and the reverse Warburg effect in stromal cells might not be mutually exclusive. Metabolism plasticity confers cancer cells potential of adaptation to complex and adverse situations and metabolic behaviour is also determined by cancer stages and types. Although hCINAP restrained OXPHOS by enhancing glycolysis in CRC, CRCSCs had not cut off the OXPHOS-based ATP supply for stromal cells, suggesting that the reverse Warburg effect and the Warburg effect concurred in CRC.

We added the results above in Supplementary Figure 3a.

(iv)Page 9, line192: Its AP5A in the text but 5 is subscript in the figures

Response:

Following the suggestion of the reviewer, we have used “AP5A” in both text and figures.

(v) Page 13, line 261: The word dramatically is too bold here - its increased for sure but I would not call it dramatic.

Response:

We have deleted the word “dramatically” in our revision.

(vi) Figures: As a general comment on all the figures, the annotations/axis labels appear blurred even on the on screen pdf version of the manuscript. This makes it hard to read. Furthermore the figure legends don't always tell the reader what the experimental conditions are (for example, what is SDAC and TIC in figure 2f? You can work it out but my feeling was that the ability to 'read' the paper would be enhanced if these conditions were defined in the legend.

Response:

As suggested by the reviewer, we have modified the annotations/axis labels in Figures and legends, and detailed the conditions in our revision. Figure 2f has been move to Supplementary Figure 6c.

(vii) Figure 5b and c: The text debating conditions in each lane are hard to read as there is no or a very small gap in the text.

Response:

We have optimized the text debating conditions in Figure 5h, i (previous Figure 5b, c) of our revision.

Reviewer #3 (Remarks to the Author):

In this manuscript, the authors present evidence to suggest a role for CINAP (AK6) in regulating colorectal cancer stemness. Patient data revealed that CINAP expression is increased in CRC tissues compared to adjacent normal mucosae. The authors subsequently functionally test the role of CINAP in HCT116 by a series of knock down and overexpression experiments. They conclude that CINAP regulates key features of cancer stemness, such as clonogenic potential, invasive capacity and therapy resistance. Mechanistically, CINAP seems to regulate LDHA activity, at least in part by facilitating LDHA phosphorylation at key residues by FGFR1. This presumably results in an increase in glycolysis and cancer stem cell phenotype. Although the manuscript contains some interesting parts, the role of FGFR1 in regulating LDHA has previously been described (REF: 21969607), hence the novelty around this section is only incremental. In addition, the choice of the cancer cell line HCT116 is not suitablefor most experiments, as it has been shown not to follow the CSC model (PMID: 9294210). They should use another CRC line to evaluate the impact of CINAP on CSC function.

Response:

As suggested by the reviewer, HCT116 cells have been reported not to follow the CSC model and are not fitting to test the effect of hCINAP on CRCSC stemness. SW480 cells were documented to follow the CRCSC model (*Cancer Cell*, 2015, PMID: 26678341), we therefore used SW480 cell line to assess the function of hCINAP on CRCSCs function. The results related to HCT116 in our primary submission were replaced in Figure 1b-d, Figure 3a,c-g, Figure 4a,c,e, Figure 5b,c,g-i, Supplementary Figure 2a, Supplementary Figure 3a-c, Supplementary Figure 4a-d, Supplementary Figure 7a-c in the revision.

Subsequently, the effect on CSCs should be delineated further. Are CSCs dying or differentiating into other lineages upon CINAP inhibition? If so, why are these cells more dependent on CINAP/LDHA/Glycolysis? These data would anchor the manuscript.

Response:

To further investigate the effect of hCINAP on CRCSCs, we used FACS analysis to detect whether CRCSCs death were induced by hCINAP depletion, and also performed quantitative real-time PCR to test whether hCINAP knockdown affected the expression of CRCSCs ($CD133^+$ colorectal cancer stem cells) differentiation markers. Meanwhile, CRCNSCs ($CD133^-$ colorectal cancer non-stem cells) and colon organoid (human normal colon stem cells) served as control in FACS and qRT-PCR analyses. The results of FACS showed that hCINAP shRNA caused more significant apoptosis in cancer cells including CRCSCs and CRCNSCs than that in organoid (Figure 6m). Generally, Cellular stress such as reactive oxygen species (ROS) induces cell apoptosis, and glucose metabolism, EMT and CSC phenotype has been verified to be associated with ROS generation (*Nature. 2012*, **485**, 661-665; *Cancer Cell. 2013*, **23**, 265-267). We therefore measured ROS level in CRCSCs, CRCNSCs and organoid, and found that hCINAP depletion increased ROS levels in CRCSCs and CRCNSCs, but not in organoid. Moreover, hCINAP knockdown induced more ROS production and apoptosis in CRCSCs than CRCNSCs, suggesting that CRCSCs were more sensitive to hCINAP inhibition (Figure 6l).

In addition, high $NADH/NAD^+$ ratio stimulates superoxide generation by complex I and alpha-ketoglutarate dehydrogenase, and LDHA converts pyruvate and NAD^+ into lactate and NADH, elevating cellular $NADH/NAD^+$ ratio. To test whether hCINAP increased $NADH/NAD^+$ ratio by enhancing LDHA activity, by using SoNar system (*Cell metabolism. 2015*, **21**, 777-789), we measured $NADH/NAD^+$ ratio in CRCSCs, CRCNSCs and organoid treated with hCINAP or control shRNA. The results exhibited that hCINAP depletion by shRNA resulted in greater increase of $NADH/NAD^+$ ratio in CRCSCs than that of CRCNSCs, and had a slight impact on $NADH/NAD^+$ ratio in organoid (Figure 6k), indicating that the increase of $NADH/NAD^+$ ratio caused by hCINAP depletion was resulted from suppressed glycolysis and promoted OXPHOS (Figure 6i-k).

Taken together, these results indicated that highly-expressed hCINAP in CRCSCs limited ROS production and cell survival, thus promoting CRC EMT and invasion. The new results were added in the revision (Figure 6h-m).

Finally, they are also encouraged to make use of inducible knock down constructs for their in-vivo experiments to rule out engraftment artifacts.

Response:

As suggested by the reviewer, we have reformed the *in vivo* mice xenograft assay by using inducible knockdown constructs (PLKO. puro tet-on system), detailed information was provided below.

See below more detailed comments:

1) The CRC patient set should be described in more details. Is there any correlation of CINAP with disease stage or any other clinical parameter? Recent identification of molecular subsets of CRC has revealed a subtype that display poor prognosis and increased invasive features. Given the wealth of publically available data around this topic, the authors should investigate CINAP expression in various datasets/subtypes.

Response:

The detailed information of CRC patients, including patient age, sex, tumor site, pathological state and grade was provided in Supplementary Table 2. And we analyzed the correlation of hCINAP expression in TCGA COAD (Colon adenocarcinoma) and READ (Rectum adenocarcinoma), compared to the normal tissues, and four stages (I-IV) in CRC patients (TCGA) by ANOVA. We found that hCINAP was significantly overexpressed in COAD and READ (Supplementary Figure 1d), while there were no differences of hCINAP expression observed in four CRC stages (Supplementary Figure 1e). In addition, we analyzed hCINAP expression differences in different CRC subtypes based on a four consensus molecular subtypes (CMSs) with distinct features (). ANOVA analysis showed that hCINAP expression was higher in CMS2 with marked activation of Wnt and Myc signaling pathways and CMS3 with evident metabolic dysregulation (Supplementary Figure 1f). Wnt signaling promotes EMT and CRCSC self-renewal and Myc enhances LDHA expression and the Warburg effect (*Clin Cancer Res. 2011*, **17**, 647-653), and metabolic imbalance in CMS3 was consistent with our results that hCINAP enhanced LDHA activity for metabolism reprogramming in CRCSCs.

Taken together, bioinformatics analyses indicate that hCINAP is overexpressed and linked with cell metabolism regulation in CRC.

Figure 1b/c should be corrected for proliferation effects.

Response:

Following the suggestion form the reviewer, cell proliferation rates were measured to rule out that migration potential was affected by excess gap of cell growth rate (Figure 7e).

2) Figure 2. An isotype control is warranted to setup gating.

Response:

As suggested, we have attached isotype controls for gating setup in the revision (Figure 2a and Supplementary Figure 5c).

The increase of CD133/Lgr5 double positive is of note but seems at odd when clonogencity is evaluated by limiting dilution. A ~8 fold increase in the fraction of CSCs only results in a modest ~1.4 fold increase in clonogenicity, this data is unclear.

Response:

In general, the percentage of cancer stem cell in overall cancer cells determines the efficiency of clonogencity in limiting dilution assay. We found that the gating setup was not strict enough in FACS analysis of the percentage CD133/LGR5 double positive CRCSCs and we had repeated FACS with a suitable gating in the revised manuscript (Figure 2a). And the approximate treatment in limiting dilution assy might leads to the disagreement of positive rate in these two experiments (LDA analysis in limiting dilution assay and student *t* test in FACS).

Minor: the mesenchymal/epithelial panel of genes in 2d is inverted.

Response:

Thanks for pointing out the mistake, and we corrected it in this revision.

3) The authors should rule out that CINAP inhibition mediates a more general cancer cell growth inhibition that CSC specific. FACS sort of CD133 pos/neg cells could be done to correlate expression of CINAP, LDHA activity etc. Indeed, stem cell genes and EMT are affected but this doesn't necessarily imply that a stem cell pool is altered.

Response:

To test whether hCINAP inhibition caused different specific effect on CRCSCs (but not on CRCNSCs), we sorted $CD133^+$ CRCSCs and $CD133^-$ CRCNSCs by FACS. And we found that hCINAP depletion led to inhibition in both CRCSCs and CRCNSCs, while CRCSCs were more sensitive to hCINAP knockdown than CRCNSCs in affecting ECAR, OCR, $NADH/NAD^+$ ratio, ROS level and apoptosis rate. Notably, hCINAP inhibition showed less impact on colon normal stem cells, indicating a promising medical intervention by targeting hCINAP (Figure 6i-m, Supplementary Figure 5f, g). In addition, $CD133^+$ CRCSCs did not express much higher levels of hCINAP and LDHA Y10 phosphorylation than $CD133^-$ CRCNSCs, while hCINAP knockdown inhibited LDHA Y10 expression in both CRCSCs and CRCNSCs (Figure 6h). However, $CD133^+$ CRCSCs possessed a higher glycolysis level than $CD133^-$ CRCNSCs (Figure 6i). The different level of glycolysis in CRCSCs and CRCNSCs is a possible cause of instinct sensitivity to hCINAP inhibition.

4) Because CINAP has been shown recently shown to regulate cancer cell growth by regulating rRNA processing, it is important to specifically demonstrate the dependency of LDHA for mediating CINAP effect on cancer stemness.

Response:

To test whether hCINAP affected CRCSC stemness by regulating LDHA activity specifically, we performed immunofluerescence assays and tumorsphere formation assays and found that LDHA depletion by shRNA inhibited EMT and tumorsphere formation efficiency of CRCSCs. In addition, rescue expression of hCINAP wild-type but not H79G mutant in CRCSCs with knocked down LDHA restored EMT program and clonogenicity ability (Figure 5d-f), suggesting that hCINAP depends on LDHA to affect CRCSC stemness.

We added these results in Figure 5 and discussed it in the revision.

For instance, can CINAP overexpression rescue LDHA KD? Conversely, normal mucosae still display CINAP expression (figure 1a) and a positive correlation between CINAP and phosphor Y10 LDHA. Are the authors anticipating gut toxicity from CINAP inhibition in healthy tissue as well? Could an organoid culture from human colon be established to test this?

Response:

Although hCINAP had a higher expression in CRC than normal tissue, hCINAP is still expressed in normal mucosae (Figure 1a, Supplementary Figure 1a-d). To test whether hCINAP inhibition would lead to gut toxicity in colon, we cultured colon organoid by isolating colon crypts from the adjacent tissue of colon cancer patients and transfected with hCINAP or control shRNA (Supplementary Figure 5b). We did not detect changes of organoid phenotype and expression of colon stem cell differentiation markers (Supplementary Figure 5b,e). Moreover, hCINAP depletion had no impact on cell death of organoid (Figure 6m), suggesting that hCINAP inhibition caused no gut toxicity in healthy tissue.

We added these results in Figure 6 and Supplementary Figure 5, and discussed it in the revision.

5) As mentioned above, in figure 7 the lack of tumor take could be a reflection of poor engraftment or low viability of engrafted cells, altogether making the clinical relevance of the study unclear. Inducible KD would significantly lift the impact of the current manuscript.

Response:

Although hCINAP depletion resulted in significant inhibition of CRC cells growth in mice xenograft model, these results might be caused by non-ideal engraftment cell status, as mentioned by the reviewer. To rule out this factor, we used a doxycycline-inducible (tet-on) knockdown system to repeat the xenograft experiment. CRCSCs stably transfected with hCINAP and control shRNA, and rescue expression of wild-type and H79G hCINAP, were inoculated in nude mice respectively. Mice were administrated with doxycycline six days later and sacrificed 12 days later (Figure 7a), and tumors were resected and measured. We found that hCINAP depletion significantly inhibited tumor growth in mice and re-expressing wild-type hCINAP, but not H79G mutant, recovered tumor growth (Figure 7a,b). Levels of LDHA Y10 phosphorylation and hCINAP in engrafted cells and tumors were detected by Western blot (Figure 7c), suggesting that hCINAP promoted CRC cells growth *in vivo* by enhancing LDHA Y10 phosphorylation, consistent with our xenograft mice data before.

REVIEWERS' COMMENTS:

Reviewer #1 (Remarks to the Author):

The authors have satisfactorily addressed this reviewer's concerns. The revised manuscript is suitable for publication in Nature Communications.

Reviewer #2 (Remarks to the Author):

I'm happy with the changes made to my comments and am happy to recommend publication.

Reviewer #3 (Remarks to the Author):

In their revised manuscript, the authors have responded well to the shared and individual concerns of each reviewer. The authors provide considerable additional data, including inducible depletion of CINAP in vivo to confirm its critical role during tumor progression.

I have few remaining suggestions, none of which should require additional experiments:

- In Figure 7g, the axis states mammosphere instead of tumorsphere (or colonosphere).

- The inducible KD in vivo is quite revealing and the rapid decrease in growth after 6 days is indeed suggestive of apoptosis rather than tumor differentiation. If the authors now have additional data at later time points after KD, including them in this paper would make it more robust. In the current setting, the therapeutical benefit still remains unclear. In other words, is it delayed growth or will interference with the cancer stem cell pool ultimately lead to tumour regression. Without this data, I would suggest the authors to be more conservative with their conclusion.

Reviewer #1 (Remarks to the Author):

The authors have satisfactorily addressed this reviewer's concerns. The revised manuscript is suitable for publication in Nature Communications.

Reviewer #2 (Remarks to the Author):

I'm happy with the changes made to my comments and am happy to recommend publication.

Reviewer #3 (Remarks to the Author):

In their revised manuscript, the authors have responded well to the shared and individual concerns of each reviewer. The authors provide considerable additional data, including inducible depletion of CINAP in vivo to confirm its critical role during tumor progression.

I have few remaining suggestions, none of which should require additional experiments:

- In Figure 7g, the axis states mammosphere instead of tumorsphere (or colonosphere).

Response:

Thanks for pointing out the mistake, and we changed “mammosphere” in Figure 7g to “tumorsphere”.

- The inducible KD in vivo is quite revealing and the rapid decrease in growth after 6 days is indeed suggestive of apoptosis rather than tumor differentiation. If the authors now have additional data at later time points after KD, including them in this paper would make it more robust. In the current setting, the therapeutical benefit still remains unclear. In other words, is it delayed growth or will interference with the cancer stem cell pool ultimately lead to tumour regression. Without this data, I would suggest the authors to be more conservative with their conclusion.

Response:

To further delineate the effect of hCINAP on CRCSCs, additional details about tumor growth at later time points (after Day 6) after hCINAP depletion by using doxycyclineat were added in the revision as follow. We also found that hCINAP depletion had a rapid effect on tumor volume augment in hCINAP knockdown and hCINAP H79G mutant rescue groups within two days (days 6-8). However, in control and hCINAP wild-type rescue groups, tumor growth was not significantly affected. Moreover, sustaining doxycycline treatment resulted in further decrement on tumor volume in hCINAP knockdown and hCINAP H79G mutant rescue groups at days 10 and 12, and had no effect on tumor growth in shRNA control and hCINAP wild-type rescue groups. These results indicate that hCINAP depletion inhibits LDHA Y10 phosphorylation in CRCSCs and induces tumor bulk degeneration *in vivo* by diminishing the colorectal cancer stem cell pool, instead of delaying tumor cells growth. And we propose hCINAP as a potent drug target in colorectal oncotherapy.

We modified Figure 7a in the revision according to the updated results.
